# Ammonia Suppresses the Antitumor Activity of Natural Killer Cells and T Cells by Decreasing Mature Perforin

**DOI:** 10.1158/0008-5472.CAN-24-0749

**Published:** 2025-03-31

**Authors:** Joanna Domagala, Tomasz M. Grzywa, Iwona Baranowska, Magdalena Justyniarska, Ryan Tannir, Agnieszka Graczyk-Jarzynka, Aleksandra Kusowska, Maria Lecka, Marcin Poreba, Klaudyna Fidyt, Katsiaryna Marhelava, Zofia Pilch, Lea K. Picard, Tomasz Wegierski, Kamil Jastrzebski, Marta Krawczyk, Marta Klopotowska, Monika Granica, Doris Urlaub, Szymon Hajduk, Alexandra Neeser, Spencer Moros, Pawel Kozlowski, Malgorzata Bobrowicz, Marta Miaczynska, Leyuan Ma, Carsten Watzl, Magdalena Winiarska

**Affiliations:** 1 Department of Immunology, Medical University of Warsaw, Warsaw, Poland.; 2 Department of Immunology, Mossakowski Medical Research Institute, Polish Academy of Sciences, Warsaw, Poland.; 3 The Raymond G. Perelman Center for Cellular and Molecular Therapeutics, Children’s Hospital of Philadelphia, Philadelphia, Pennsylvania.; 4 College of Arts and Sciences, University of Pennsylvania, Philadelphia, Pennsylvania.; 5 Doctoral School, Medical University of Warsaw, Warsaw, Poland.; 6 Faculty of Chemistry, Wroclaw University of Science and Technology, Wroclaw, Poland.; 7 Faculty of Medicine, Wroclaw University of Science and Technology, Wroclaw, Poland.; 8 Department for Immunology, Leibniz Research Centre for Working Environment and Human Factors (IfADo) at TU Dortmund, Dortmund, Germany.; 9 International Institute of Molecular and Cell Biology, Warsaw, Poland.; 10 Doctoral School of Translational Medicine, Mossakowski Medical Research Institute, Polish Academy of Sciences, Warsaw, Poland.; 11 Central Laboratory, University Clinical Centre of the Medical University of Warsaw, Warsaw, Poland.; 12 Department of Pathology and Laboratory Medicine, Perelman School of Medicine, University of Pennsylvania, Philadelphia, Pennsylvania.; 13 Center for Cellular Immunotherapies, University of Pennsylvania, Philadelphia, Pennsylvania.; 14 Abramson Cancer Center, University of Pennsylvania, Philadelphia, Pennsylvania.

## Abstract

Immunotherapy revolutionized cancer treatment in the last decade. Both NK cells and T cells are key components of host immunity against malignant cells that are being extensively investigated in the field of cancer immunotherapy. While approaches have been developed to improve the antitumor activity of NK and T cells, the tumor microenvironment remains an obstacle to effective NK and T‐cell–based therapies. Here, we demonstrated that cancer‐conditioned medium suppresses the antitumor activity of NK cells. Ammonia, a by‐product of cancer cell metabolism, accumulated in cancer‐conditioned medium and in the tumor microenvironment, and impaired the cytotoxicity of NK cells as well as the efficacy of antibody‐based and chimeric antigen receptor (CAR) NK and CAR T‐cell–based therapies *in vitro*. Ammonia induced NK and T‐cell dysfunction by decreasing the amount of mature perforin in secretory lysosomes, which was dependent on its lysosomotropic features and ability to increase pH in acidic compartments. These findings demonstrate that, in addition to its previously described role in promoting tumor growth as a source of nitrogen for tumor biomass, ammonia promotes tumor immune escape by inhibiting both NK and CAR T‐cell cytotoxicity.

**Significance:**

Ammonia is elevated in the tumor microenvironment and functions as an immunoinhibitory metabolite in cancer by reducing perforin levels, inhibiting NK and T‐cell–mediated immunity and limiting the efficacy of immunotherapies.

## Introduction

NK cells constitute a crucial component of the antitumor immune response (1). Due to their potent cytotoxicity against malignant cells and relatively low sensitivity to inhibitory signals in cancer, they are extensively studied in the field of immunotherapy. Beyond their natural cytotoxic potential, NK cells can kill tumors through a mechanism of antibody-dependent cell-mediated cytotoxicity (ADCC) by engaging with monoclonal antibodies, including clinically available antibodies targeting CD20 (rituximab, RTX), CD38 (daratumumab), and HER2 (trastuzumab; ref. 2). T cells are another immune cell population extensively studied in cancer treatment and have been introduced into clinics with great success (3). T-cell–based immunotherapies include immune checkpoint inhibitors, bispecific T-cell engagers, and tumor-infiltrating lymphocyte therapies (3). Additionally, NK cells, as well as T cells, can be used in adoptive cell therapies as chimeric antigen receptor (CAR)–modified cells (4). Nonetheless, multiple factors secreted by cancer cells and other types of cells in the tumor microenvironment (TME) suppress their activity, including not only protein factors such as cytokines but also different metabolites (5, 6).

Dysregulation of cellular metabolism is one of the hallmarks of cancer (7). For instance, cancer cells rely on glycolysis and glutaminolysis to a much greater extent than healthy cells (8). This dysregulation results in a specific profile of metabolites in the TME (9). Although in normal tissue products of metabolism are excreted, impaired tumor tissue architecture and abnormal vascularization lead to their accumulation in the TME. This includes lactate and ammonia, which promote tumor growth by serving as a source of energy and nitrogen, respectively (10, 11).

Recently, ammonia concentrations have been demonstrated to be substantially increased in the TME, in which immune cells interact with cancer cells. Previous studies also revealed that ammonia may regulate the immune response by suppressing the activity of macrophages, dendritic cells, and neutrophils (12–15). Furthermore, ammonia has been identified as a driver of T-cell exhaustion and a regulator of the development of memory T cells (16–18). However, whether ammonia impairs the cytotoxic functions of immune cells, including NK and cytotoxic T cells, remains unknown.

In this study, we demonstrated that cancer-conditioned medium suppresses the antitumor activity of NK cells, and this effect is dependent on glutamine. We identified that ammonia accumulates in cancer-conditioned medium and tumor interstitial fluid (TIF) in mice and inhibits the cytotoxic activity of NK and T cells by decreasing the amount of mature perforin in secretory lysosomes. Collectively, our findings suggest that ammonia plays a role as an inhibitory metabolite, suppressing the cytotoxic activity of NK and T cells.

## Materials and Methods

### Cell culture

Cells were cultured in RPMI 1640 (Raji, RRID:CVCL_0511; Daudi, RRID:CVCL_0008; Ramos, RRID:CVCL_0597; MCF7, RRID:CVCL_0031; Nalm6, RRID:CVCL_0092; Gibco) or DMEM (E0771, RRID:CVCL_GR23; EMT6, RRID:CVCL_1923; U87; Sigma-Aldrich) supplemented with 10% FBS (HyClone) and 1% penicillin–streptomycin solution (Gibco) in a humidified atmosphere containing 5% CO_2_ at 37°C. Multiple myeloma cell lines (MM1s, RRID:CVCL_8792; RPMI 8226, RRID:CVCL_0014; H929, RRID:CVCL_1600) were cultured in RPMI 1640 medium additionally supplemented with 50 mmol/L 2-mercaptoethanol (1,000x; Gibco) and 100 mmol/L sodium pyruvate (100x; Gibco). The NK-92 cell line was maintained in X-VIVO 20 medium (Lonza) supplemented with 5% human serum (Sigma-Aldrich). All cell lines were obtained from ATCC, and further authentication testing was not conducted. Cells were regularly tested for *Mycoplasma* contamination using the PCR method. The time between thawing the cells and use in the described experiments was no longer than 1 month. Cell media were changed every 48 hours. For 3D cell culture, 5 × 10^3^ 4T1 cells were seeded in a 48-well plate on lactose dehydrogenase elevating virus (LDEV)-free Matrigel (Corning) in RPMI supplemented with 5% FBS, 1% penicillin–streptomycin, and 2% Matrigel. Cells were cultured for 14 days.

### Conditioned medium generation

Conditioned medium was collected after 48 hours of culture of various cancer cell lines. Cells were seeded into six-well plates at the following densities: Raji, Daudi, Ramos, HCC1806 (RRID:CVCL_1258), 4T1 (RRID:CVCL_0125), EMT6, MM1s, H929, RPMI 8226, and T cells at 1 × 10^6^ cells/mL; E0771 at 1.5 × 10^6^ cells/mL; and L929 at 0.2 × 10^6^ cells/mL. The collected conditioned medium was centrifuged at 1,500 *g* for 4 minutes and then filtered through a 0.45 μm pore filter. Subsequently, to assess the NK cells’ natural cytotoxicity, K562 and NK cells obtained from healthy donors were incubated for 4 hours in the presence of the conditioned medium. An Amicon Ultra-0.5 Centrifugal Filter Unit Ultracel-3 was used according to the manufacturer’s protocols to separate a small-molecule fraction (<3 kDa) and a large-molecule fraction (>3 kDa) from the conditioned medium.

### NK cell isolation

Peripheral blood mononuclear cells (PBMC) were isolated from buffy coats of healthy donors by gradient centrifugation using Ficoll (Sigma-Aldrich). Buffy coats were purchased from the Regional Blood Centre in Warsaw; the procedure was approved by the Local Bioethics Committee (approval number: AKBE/61/2021). Primary NK cells were isolated from PBMCs by immunomagnetic negative selection using the EasySep Human NK Cell Enrichment Kit (STEMCELL Technologies) according to the manufacturer’s protocols. NK cells were cultured in full RPMI 1640 medium unless otherwise described for specific experimental procedures. For conjugate and detachment assays, human NK cells were isolated from PBMCs with the Dynabeads Untouched Human NK Cells Kit according to the manufacturer’s instructions (Invitrogen). Isolated NK cells were seeded in 96-well round-bottom plates (Nunc) at a density of 1 to 1.5 × 10^6^ cells/mL with 0.5 × 10^6^/mL feeder cells irradiated with 30 Gy before use (K562-mbIL15-41BBL) in a culture medium supplemented with 200 U/mL of IL2 and 100 ng/mL of IL21. A medium exchange (50% old medium removed and fresh medium with 100 U/mL IL2 added) was performed after 4 days. On day 8, NK cells were restimulated by the addition of 0.5 × 10^6^/mL feeder cells and 200 U/mL IL2. In the following weeks, depending on cell density, medium was exchanged, or cells were split to a density of 1.5−2 × 10^6^/mL with medium containing 100 U/mL IL2. From day 14 onwards, 2.5 ng/mL IL15 was added when cells were split or a medium exchange was performed. Starting at week 3, the NK cells were used for experiments.

### T-cell isolation

PBMCs were isolated from buffy coats of healthy donors by gradient centrifugation using Ficoll (Sigma-Aldrich). Buffy coats were purchased from the Regional Blood Centre in Warsaw; the procedure was approved by the Local Bioethics Committee (approval number: AKBE/61/2021) and was performed in accordance with the Declaration of Helsinki. All donors signed written informed consent. Primary T cells were isolated from PBMCs by immunomagnetic negative selection using the EasySep Human T Cell Enrichment Kit (STEMCELL Technologies) according to the manufacturer’s protocols. T cells were cultured in full RPMI 1640 medium with 100 U/mL of recombinant human IL2 (PeproTech) unless otherwise described for specific experimental procedures.

### Animal studies

All *in vivo* experiments were performed with 8- to 12-week-old female mice obtained from Charles River Laboratories, which were bred at the animal facility of the Department of Immunology, Medical University of Warsaw. All experiments were performed in accordance with the guidelines and were approved by the Second Local Ethics Committee for Animal Experimentation, Warsaw University of Life Sciences (numbers: WAW2/175/2018, WAW2/111/2019, WAW2/090/2020, WAW2/005/2021, WAW2/074/2021, WAW2/019/2019, WAW2/108/2021, WAW2/155/2022, WAW2/022/2022, WAW2/122/2023).

### *In vivo* tumor models

Experiments were performed on NOD/SCID gamma (NSG; RRID:IMSR_CRL:394), SCID, BALB/c (RRID:IMSR_APB:4790), or C57BL/6 (RRID:MGI:2159769) female mice. NSG mice were inoculated subcutaneously with human cell lines: 2 × 10^6^ Raji, MDA-MB-231, or 3 × 10^6^ MCF7 cells. In the case of the experiments involving MCF7 cells, slow-release pellets containing 17β-estradiol (Innovative Research of America) were implanted subcutaneously 4 days before tumor cell inoculation. SCID mice were inoculated subcutaneously with 3 × 10^6^ MM.1s cells. BALB/c mice were inoculated into the left mammary fat pad with murine cell lines: 0.37 × 10^6^ 4T1 cells or subcutaneously with 0.37 × 10^6^ EMT6 cells. C57BL/6 mice were inoculated subcutaneously with 0.37 × 10^6^ E0771 cells. Tumor volume was calculated according to the formula mm^3^= [width^2^ (mm) × length (mm)]/2. Mice were sacrificed when the tumor diameter reached 15 mm in at least one dimension. Tumors were used for TIF and subcutaneous fluid (SCF) isolation.

### Tumor interstitial fluid collection

TIF was isolated from tumors not exceeding 1,500 mm^3^. Mice were anesthetized by administering 10 mg of ketamine and 1.5 mg of xylazine per 100 g of body weight. TIF was isolated using a UF-1-2 *In Vivo* Ultrafiltration Sampling Probe (BASi, MF-7027). The probe was implanted centrally into the tumor for 2 hours to collect TIF. The SCF of healthy tissue was extracted by a probe implanted under the skin on the opposite side of the tumor. High-molecular-weight compounds were excluded from the analytes using the filtration membrane. Following ultrafiltration, approximately 10 μL of TIF and 12 to 18 μL of SCF were obtained. Ammonium was immediately measured using a Dimension Ammonia assay (Siemens).

### *In vivo* therapy with RTX and intratumoral administration of NH_4_Cl

Experiments were performed on BALB/c, BALB/c SCID, and NOD.Cg-*Prkdc*^*scid*^*Il2rg*^*tm1Wjl*^/SzJ (NSG) mice. Mice were inoculated subcutaneously with 2 × 10^6^ Raji cells in 50% Matrigel Growth Factor Reduced (Corning, LifeSciences). Rituximab-treated mice were injected intraperitoneally with 10 mg/kg RTX three times per week for 2 consecutive weeks. NH_4_Cl-treated mice were injected intratumorally with 50 mmol/L NH_4_Cl daily for 2 consecutive weeks. Tumor volumes were measured three times a week, starting from day 7 of the experiment. Tumor volume was calculated according to the following formula: mm^3^= [width^2^ (mm) × length (mm)]/2. Mice were sacrificed when the tumor diameter reached 15 mm in at least one dimension.

### Ammonia measurement

Ammonia concentration was measured in tumor-conditioned medium collected after 48 hours of incubation or in TIF and SCF immediately after collection. Ammonia concentration in conditioned medium was measured using the Dimension Ammonia assay (Siemens). High-molecular-weight compounds were excluded from the analytes by a filtration membrane in the process of TIF and SCF isolation. The 0.5 mL conditioned medium was centrifuged (5 minutes, 500 *g*) and filtered through an Amicon Ultra-0.5 Centrifugal Filter unit (Merck). Ammonia in TIFs and SCFs was then quantified using the Dimension Ammonia assay (Siemens) by the Laboratory of the Central Clinical Hospital of the University Clinical Center of Warsaw, Medical University of Warsaw, Poland.

### Antibodies

Fluorophore-conjugated antibodies specific for cell-surface antigens and cytokines were as follows: anti-CD16, clone 3G8 (BioLegend, RRID:AB_2563803); anti-CD56, clone B159 (BD Biosciences, RRID:AB_395906); anti-NKp30, clone p30-15 (BD Biosciences, RRID:AB_2738170); anti-NKp44, clone p44-8 (BD Biosciences); anti-NKp46, clone 9E2 (BD Biosciences, RRID:AB_398653); anti-NKG2D, clone 1D11 (BD Biosciences, RRID:AB_11153309); anti-CD95, clone DX2 (BioLegend, RRID:AB_314550); anti-CD178, clone NOK-1 (BD Biosciences, RRID:AB_2738714); anti-CD107a, clone H4A3 (BD Biosciences, RRID:AB_396135); anti-CD3, clone OKT3 (BioLegend, RRID:AB_2619696); anti-perforin, clone δG9 (BioLegend, RRID:AB_2566204, and BD Biosciences, RRID:AB_396419); anti-perforin, clone D48 (BioLegend, RRID:AB_2616860); anti–granzyme B, clone GB11 (BD Biosciences, RRID:AB_1645488); anti-IFNγ, clone 25723.11 (BD Biosciences, RRID:AB_2264629); anti-TNFα, clone MAb11 (BD Biosciences, RRID:AB_397219); and anti-DYKDDDDK (FLAG tag), clone L5 (BioLegend, RRID:AB_2536846). Antibodies were used at a concentration recommended by the manufacturers.

### Flow cytometry analysis

Flow cytometry was performed on FACSCanto II (BD Biosciences), Fortessa X-20 (BD Biosciences), LSRFortessa (BD Biosciences), or FACSCalibur (BD Biosciences) operated by FACSDiva software (RRID:SCR_001456). For data analysis, FlowJo version 10.6.1 software (Tree Star, RRID:SCR_008520) or BD FACSDiva software (BD Biosciences) was used. For cell surface staining, cells were stained with Zombie NIR or Zombie Aqua Fixable Viability Kit (BioLegend) for 15 minutes at room temperature, washed with FACS buffer (PBS; 1% BSA, 0.01% sodium azide), blocked on ice with 5% normal rat serum in FACS buffer, and then incubated for 30 minutes on ice with fluorochrome-labeled antibodies listed above. After washing in FACS buffer, cells were immediately analyzed.

### Intracellular staining

Cells were seeded at a density of 0.2 × 10^6^ NK and 0.1 × 10^6^ K562 cells [effector:target (E/T) ratio 2:1] per well of a U-bottom plate and coincubated for 4 hours at 37°C in the presence of NH_4_Cl and 1 μL GolgiPlug (BD Biosciences). After that time, cells were washed three times with 200 μL PBS and centrifuged (5 minutes, 500 *g*). Cells were stained with a viability dye (Zombie NIR Fixable Viability Kit), followed by surface anti-CD56 antibody staining, and fixed with 100 μL/well Fixation Solution (BD Biosciences, RRID:AB_2869005). Next, cells were washed twice with 200 μL Permeabilization Buffer (BD Biosciences) and centrifuged (5 minutes, 500 *g*). Subsequently, cells were stained with IFNγ-APC and TNFα-PE (BD Biosciences) for 30 minutes at room temperature. In the last step, cells were centrifuged (5 minutes, 500 *g*), resuspended in 200 μL PBS, and analyzed using a BD FACSCanto II (BD Biosciences). For perforin staining, cells were seeded at a density of 0.2 × 10^6^ NK and 0.1 × 10^6^ K562 cells (E/T ratio 2:1) per well of a U-bottom plate and coincubated for 4 hours at 37°C in the presence of NH_4_Cl. After that time, cells were washed three times with 200 μL of PBS and centrifuged (5 minutes, 500 *g*). Cells were stained with viability dye (Zombie NIR Fixable Viability Kit), followed by surface anti-CD56 antibody staining, and fixed with 100 μL/well of Fixation Solution (BD Biosciences). Next, cells were washed twice with 200 μL of Permeabilization Buffer (BD Biosciences) and centrifuged (5 minutes, 500 *g*). Subsequently, cells were stained with perforin-PE (BD Biosciences) for 30 minutes at room temperature. In the last step, cells were centrifuged (5 minutes, 500 *g*), resuspended in 200 μL of PBS, and analyzed using the BD FACSCanto II (BD Biosciences).

### qRT-PCR

NK cells were isolated from PBMCs using the EasySep Human NK Cell Enrichment Kit (STEMCELL Technologies) and primed with IL2 (200 U/mL) and IL15 (10 ng/mL) for 24 hours. Following priming, the cells were seeded in a culture medium containing IL2 and IL15 cytokines and 2.5, 5, or 10 mmol/L NH_4_Cl. Twenty-four hours after ammonia treatment, the cells were harvested, washed with PBS, and processed for mRNA isolation. To obtain a primary T-cell population, the PBMCs were stimulated with PHA-L for 3 days. After that, stimulated T cells were seeded in culture medium containing IL2 (100 U/mL) and 2.5 mmol/L or 5 mmol/L NH_4_Cl for 24 or 48 hours. Next, the T cells were harvested, washed with PBS, and processed for mRNA isolation. Total RNA from NK cell/T-cell samples after ammonia treatment was isolated using the RNeasy Mini Kit (Qiagen) and RNase-Free DNase Set (Qiagen) according to the manufacturer’s instructions. Next, using 1 μg of RNA, cDNA synthesis was performed using the Maxima First Strand cDNA Synthesis Kit for qRT-PCR (Thermo Fisher) and a Bio-Rad thermocycler. To perform the qRT-PCR, the PowerUp SYBR Green Master Mix (Applied Biosystems) was used together with specific perforin (PRF-1) primers (forward: GGAGTGCCGCTTCTACA, reverse: CTGGGTGGAGGCGTTGAA). The reaction was run using a 7500 Fast Real-Time PCR System (Applied Biosystems). The relative PRF-1 mRNA level was analyzed using the comparative Ct (ΔCt) method relative to the mean of *TBP* (forward: GCACAGGAGCCAAGAGTGA, reverse: GTTGGTGGGTGAGCACAAG) and *SDHA* housekeeping genes (forward: GCATTTGGCCTTTCTGAGGC, reverse: CTCCATGTTCCCCAGAGCAG).

### NK natural cytotoxicity and ADCC

K562 cells were stained with the CellTrace CFSE Cell Proliferation Kit (Thermo Fisher Scientific) for 10 minutes at 37°C. K562 cells were seeded in U-bottom 96-well plates at a density of 0.5 × 10^5^ K562 cells per well and incubated either with 0.25 × 10^6^ NK cells (E/T ratio 5:1) or 0.1 × 10^6^ NK cells (E/T ratio 2:1) in the presence of NH_4_Cl or non-diluted conditioned medium. For ADCC experiments, RTX at a final concentration of 100 μg/mL or daratumumab at a final concentration of 1 μg/mL was added. The plate was incubated for 4 hours at 37°C. Subsequently, cells were stained with 4 μg/mL propidium iodide, and viability was analyzed with a BD FACSCanto II (BD Biosciences) as a percentage of propidium iodide–positive carboxyfluorescein succinimidyl ester (CFSE)-positive cells.

### RTCA cytotoxic assay

The xCELLigence real-time cell analysis (RTCA) system (ACEA Biosciences) was used to monitor the viability of the MCF7 cells during the coincubation with the NK cells and NH_4_Cl. MCF7 cells were seeded on 16-well E-Plates (ACEA Biosciences) at a cell density of 3 × 10^4^ per well in 150 μL of DMEM and monitored for 24 hours. Next, the medium from the E-Plates with target cells was removed and replaced with NK cells added at an E/T ratio of 2:1, together with trastuzumab (final concentration 10 μg/mL) and NH_4_Cl (Sigma-Aldrich). The cells were monitored with the RTCA system for the next 12 to 20 hours. The analysis of the data was performed using RTCA Software Pro (ACEA Biosciences), and results are presented as a normalized cell index.

### Luciferase-based killing assay

Luciferase-based assays were used to determine the cytotoxicity of CD19-CAR-NK92 cells against Raji-luc cells. Target cells were seeded onto U-bottom 96-well black plates at a density of 0.25 × 10^5^ cells per well in 50 μL of DMEM. NK cells were added at a density of 0.25 × 10^5^ (E/T ratio 1:1) in 50 μL of DMEM together with 100 μL NH_4_Cl (for a final concentration in the range of 1.25–10 mmol/L). The plate was incubated for 18 hours at 37°C in a humidified atmosphere with 5% CO_2_. The following day, 100 μL of each sample was transferred to a white 96-well plate, and 100 μL of the mix of Bright-Glo Luciferase Assay (Promega) reagents was added to each well. The plate was incubated for 5 minutes in darkness; the bioluminescence signal was detected using a VICTOR Plate Reader (PerkinElmer).

### Conjugates assay

A total of 6 × 10^6^ NK cells were stained with CellTracker Deep Red Dye (Thermo Fisher Scientific) for 10 minutes at 37°C. At the same time, 12 × 10^6^ K562 cells were stained with the CellTrace CFSE Cell Proliferation Kit (Thermo Fisher Scientific) for 10 minutes at 37°C. Next, the cells were washed thoroughly in PBS and resuspended in 3 mL of cell medium. NK and K562 cells were divided and incubated with NH_4_Cl (Sigma-Aldrich) for 2 hours at 37°C. This was followed by co-incubation of NK cells (0.375 × 10^6^) with K562 cells (0.75 × 10^6^) in a total of 100 μL. The reaction was stopped by brief vortexing and the addition of 100 μL of 4% paraformaldehyde (PFA) at different time points (0, 5, 15, 30, and 45 minutes). Cells were analyzed immediately using a BD LSRFortessa (BD Biosciences), and conjugates were determined as double-positive events.

### Detachment assay

NK and K562 cells were stained using Deep Red and CFSE dyes, respectively, as described before. NK and K562 cells were divided and incubated with NH_4_Cl (Sigma-Aldrich) for 2 hours at 37°C. To form initial conjugates, 0.6 × 10^6^ NK cells were mixed with 1.2 × 10^6^ K562 cells in a total volume of 600 μL in 50 mL Falcon tubes, centrifuged (300 *g*, 1 minute), and coincubated for 30 minutes at 37°C. Then, conjugate formation was stopped by briefly vortexing the cells and adding 15 mL of cell medium. The cells were then distributed to 15 mL Falcon tubes with 2.5 mL of diluted conjugates each and were incubated while rotating at 37°C, allowing the NK cells to detach but preventing the formation of new conjugates. At different time points (0, 10, 20, 40, 60, and 90 minutes), the reaction was stopped by the addition of 2.5 mL of 4% PFA. Cells were analyzed immediately using a BD LSRFortessa (BD Biosciences), and conjugates were determined as double-positive events.

### Degranulation assay

NK cells (0.2 × 10^6^/well) and K562 cells (0.1 × 10^6^/well, E/T ratio 2:1) were coincubated in a U-bottom plate for 4 hours at 37°C in the presence of NH_4_Cl and anti-CD107a antibody. Subsequently, cells were stained with a viability dye (Zombie NIR Fixable Viability Kit) and NK cell surface marker antibodies (anti-CD56 and anti-CD3). After that, cells were analyzed using a BD FACSCanto II (BD Biosciences).

### Serial degranulation assay

K562 cells were stained with the CellTrace CFSE Cell Proliferation Kit (Thermo Fisher Scientific) for 10 minutes at 37°C. Next, 0.2 × 10^6^ NK and 0.1 × 10^6^ K562 (E/T ratio 2:1) cells per well were seeded and coincubated for 2 hours at 37°C in the presence of NH_4_Cl and anti-CD107a-PE antibody. After that time, cells were washed, and fresh NH_4_Cl was added together with anti-CD107a-BV421 antibody and incubated for 4 hours at 37°C. Cells were analyzed using the BD FACSCanto II (BD Biosciences). Serial degranulation was defined as double-positive events (CD107a-PE^+^ and CD107a-BV421^+^).

### Lentiviral modification of NK-92 cells with CARs

NK-92 cells were transduced with pSEW-CD19 (FMC63)-CH3-IgG1-CD28-CD3z (CD19 CAR) or atezolizumab-based anti–PD-L1 CAR (19, 20). To produce CD19 or PD-L1 CAR viral particles, HEK293T cells (RRID:CVCL_0063) were seeded in 10 cm plates and transfected using a polyethyleneimine transfection protocol simultaneously with CAR-encoding plasmids: vesicular stomatitis virus G (VSV-G) envelope–expressing plasmid pMD2.G (RRID:Addgene_12259) and lentiviral packaging plasmid psPAX2 (RRID:Addgene_12260). After 48 hours, the lentivirus-containing supernatant was harvested, filtered through a 0.45 μm pore size filter, and concentrated by overnight centrifugation at 2,500 × *g* at 4°C. The culture medium from the NK-92 cells was replaced with concentrated lentiviral supernatant supplemented with 15 μg/mL protamine sulfate and 6 μmol/L BX-795. After 1 hour of spinoculation (750 × g at 25°C), the NK-92 cells were kept overnight in an incubator. The next day, the viral supernatant was replaced with a fresh portion of complete culture X-VIVO 20 medium (Lonza) supplemented with 200 U/mL IL2 (PeproTech). The CAR expression on the surface of the NK-92 cells was evaluated by flow cytometry 48 to 72 hours after transduction.

### ELISA

NK cells, together with K562 cells, were treated with NH_4_Cl for 4 hours at 37°C. Subsequently, the cells were extensively mixed and centrifuged, and the supernatants were collected. They were analyzed using the Human Perforin ELISA kit (MABTECH) according to the manufacturer’s protocol and measured using the PerkinElmer Multimode Plate Reader EnVision. This kit uses two types of anti-perforin antibodies: Pf-344, which recognizes an epitope after perforin monomers undergo pore formation, and Pf-80, which recognizes a conformational epitope accessible at acidic but not neutral pH.

### Western blotting

A total of 3 × 10^6^ NK cells or 4 × 10^6^ T cells per well were seeded on a six-well plate and incubated for 4 hours at 37°C in the presence of NH_4_Cl. Afterward, cells were suspended in ice-cold lysis buffer [150 mmol/L NaCl, 1% Triton X-100, 50 mmol/L Tris-HCl (pH 8.0)] or RIPA Lysis and Extraction Buffer (Thermo Fisher Scientific) supplemented with protease inhibitors (Roche) and incubated on ice for 15 minutes. The lysates were centrifuged for 10 minutes at 14,000 *g* at 4°C; supernatants were transferred to new tubes. Total protein concentration was measured using a bicinchoninic acid assay. Five to thirty micrograms of the protein lysate was mixed with four times concentrated loading Laemmli buffer [0.125 mol/L Tris (pH 6.9), 4% SDS, 10% dithiothreitol (DTT), 20% glycerol], and samples were boiled for 5 minutes at 95°C. Proteins were separated using 4% to 12% Bis-Tris NuPAGE gels (Life Technologies) or 8% or 12% SDS-PAGE gels. They were next transferred to a polyvinylidene difluoride membrane that was blocked with 5% nonfat dry milk or 5% BSA (Sigma-Aldrich) for 1 hour. Membranes were incubated overnight at 4°C with perforin, granzyme B, or granzyme H antibodies. Total perforin was detected with Abcam (#ab47225) antibody; mature and immature forms of perforin were detected with Mabtech (#3465-6-250, RRID:AB_907370) antibody. Granzyme B was detected with BioLegend (#674602) antibody. Granzyme H was detected with Cell Signaling Technology (#99695, RRID:AB_2565266) antibody. Subsequently, membranes were washed and incubated with horseradish peroxidase–conjugated secondary antibodies for 1 hour at room temperature. β-Actin antibody (#A2228, Sigma-Aldrich, RRID:AB_476697) was used as a loading control. For protein detection, SuperSignal West Pico PLUS Substrate (#34580, Thermo Fisher Scientific) or SuperSignal West Femto Maximum Sensitivity Substrate (#34096) was used. The signal was detected with the ChemiDoc Imaging System (Bio-Rad).

### Live-cell imaging with reporter cells

To investigate killing mechanisms, 0.2 × 10^5^/mL HeLa-CD48 cells that stably express NES-ELQTD-GFP-T2A-NES-VGPD-mCherry and CD48 were seeded onto a silicon–glass microchip and left to adhere overnight. Fluorescent reporters allow measurement of granzyme B (NES-RIEADS-mCherry) and caspase-8 (NES-ELQTD-GFP) activity shortly after NK cells attached to the target cell. The following day, 0.1 × 10^4^/mL NK cells were added to the microchip together with NH_4_Cl and placed into the incubation chamber (37°C, 5% CO_2_). Time-lapse live-cell microscopy was immediately started using a ZEISS Axio Observer Z1 7 microscope. EGFP and mCherry were excited using the Colibri 7 LED module 475 (filter set 90 HE LED) and 567 (filter set 91 HE LED), respectively. Images were acquired every 3 minutes for 15 to 17 hours using a Hamamatsu ORCA-Flash4.0 camera. Images were analyzed using ImageJ software.

### Serial killing

To evaluate NK cells’ serial killing ability, 0.5 × 10^6^/mL K562 cells were seeded onto a silicon–glass microchip, together with the viability dye 2.5 μmol/L SYTOX Blue (Thermo Fisher Scientific). Subsequently, 0.375 × 10^6^/mL NK cells were added together with NH_4_Cl and placed into the incubation chamber (37°C, 5% CO_2_). The analysis required tracking a randomly selected single NK cell and assessing its interaction with a target cell. Target cell viability was determined based on the intensity of the fluorescence dye SYTOX Blue, which easily penetrates cells with damaged cell membranes and stains cell nuclei. Time-lapse, live-cell microscopy was immediately started using a ZEISS Axio Observer Z1 7 microscope. Images were acquired every 3 minutes for 15 to 17 hours using a Hamamatsu ORCA-Flash4.0 camera. Images were analyzed using ImageJ software. Results were presented as a diagram showing the serial killing of NK cells.

### Microscopy

For the analysis of the lysosomal content, NK cells were incubated with NH_4_Cl for 12 hours. Then, LysoTracker Deep Red (Thermo Fisher Scientific) was added for 30 minutes at a final concentration of 50 nmol/L. Nuclei were stained with Hoechst (H1399; Thermo Fisher Scientific) at a final concentration of 5 μg/mL. Cells were imaged using Opera Phenix live cell microscopy (PerkinElmer). Images were analyzed using Harmony 4.9 software (PerkinElmer). At least 10 fields were analyzed for each of the four NK cell donors. For lysosomal-associated membrane protein 1 (LAMP1) and CD63 analysis, NK cells were incubated with NH_4_Cl for 12 hours. Then, cells were rinsed twice for 5 minutes with ice-cold PBS and fixed with 3% PFA in PBS for 12 minutes at room temperature, followed by simultaneous permeabilization with 0.1% (w/v) saponin and blocking with 0.2% (w/v) fish gelatin in PBS for 10 minutes. They were further incubated with appropriate primary and secondary antibodies in 0.01% (w/v) saponin and 0.2% fish gelatin in PBS for 30 minutes each. The mouse anti-CD63 antibody (mAb, #H5C6, RRID:AB_2572565) developed by J.T. August/J.E.K. Hildreth was obtained from the Developmental Studies Hybridoma Bank, developed under the auspices of the National Institute of Child Health and Human Resources and maintained by the University of Iowa, Department of Biology, Iowa City, IA. Rabbit anti-LAMP1 (L-1418, 1:400; RRID:AB_477157) was from Sigma-Aldrich. Secondary antibodies used for immunofluorescence were as follows: Alexa Fluor 488–, 555–, and 647–conjugated anti-mouse IgG and anti-rabbit IgG (Thermo Fisher Scientific). All secondary antibodies were diluted 1:10,000. Airyscan imaging was performed with a confocal laser scanning microscope, ZEISS LSM 800, equipped with a Plan-Apochromat 63×/1.40 NA oil objective and an Airyscan detection unit, using Immersol 518 F immersion medium (ZEISS). Detector gain and pixel dwell times were adjusted for each dataset, keeping them at their lowest values to avoid saturation and bleaching effects. ZEN Blue 2.3 (version 2.3.69.1003) software (ZEISS) was used for image acquisition. The Airyscan processing module with default settings was used to process the obtained data. Pictures were assembled in Photoshop (Adobe) with only linear adjustments of contrast and brightness.

For imaging with pH-resistant and pH-sensitive lysosomal dyes, NK-92 cells (RRID:CVCL_2142) were sequentially loaded with LysoPrime Green and pHLys Red (DOJINDO Laboratories) for 30 minutes in PBS solution, then incubated with increasing concentrations of NH_4_Cl in the Live Cell Imaging Solution (Thermo Fisher Scientific), transferred to a 96-well PhenoPlate (Revvity), and allowed to settle down. Cells were imaged on a ZEISS LSM 710 confocal microscope with an Apochromat 63×/1.40 oil objective for 45 seconds at 0.2 Hz. The pH-resistant probe and the pH-sensitive probe were excited with the 458 and 543 nm lasers, respectively, and the emission light at 465 to 538 nm and 551 to 618 nm, respectively, was collected through an open pinhole on the photomultiplier tubes. The 458 nm laser light was simultaneously collected on a transmitted light photomultiplier tube. At least three fields of view were chosen to acquire time lapses for each condition. The collected images were time-averaged, and regions of interest were marked on lysosome-like structures that contained the LysoPrime Green signal (pH-resistant probe) using ImageJ. The pHLys Red signal was divided by the LysoPrime Green signal in each region of interest, and the resulting ratios were averaged for each field of view. The experiment was repeated three times yielding similar decreases in ratio values with increasing concentrations of NH_4_Cl.

### Perforin proteolysis assay

Cathepsin B and L were expressed and purified as described elsewhere (21, 22). The catalytic activity of cathepsin L and B was confirmed by measuring fluorescence from the substrate Cbz-Phe-Arg-AMC. Both proteases were prepared at 1 to 100 nmol/L concentrations in two buffer solutions, without any preincubation: 100 mmol/L sodium acetate, 10 mmol/L sodium chloride, and 10 mmol/L DTT at pH 5.0 and 100 mmol/L HEPES, 10 mmol/L sodium chloride, and 10 mmol/L DTT at pH 7.4. Perforin (200 ng) was added to each cathepsin solution and incubated for 30 minutes. Granzyme B was purchased from Creative Enzymes (NATE-1622), and its catalytic activity was validated with the Ac-IETD-AFC fluorogenic substrate. Granzyme B solutions were prepared in two buffer conditions: one containing 20 mmol/L Tris-HCl, 100 mmol/L NaCl, 1 mmol/L EDTA, and 0.1% Triton X-100 at pH 8.0 and the other with 10% w/v sucrose, 20 mmol/L PIPES, 10 mmol/L NaCl, and 1 mmol/L EDTA at pH 7.4. Perforin (100 ng) was added to each granzyme B solution and incubated for 4 hours. All cathepsin and granzyme samples were treated with reducing loading buffer and heated at 95°C for 5 minutes. Proteins were separated by SDS-PAGE on 4% to 12% Bis-Tris gels at 200 V for 25 minutes and then transferred onto 0.2 µm nitrocellulose membranes at 10 V for 60 minutes. Membranes were blocked in 5% BSA in Tris-buffered saline with Tween 20 (TBST) for 1 hour at room temperature, followed by an overnight incubation at 4°C with anti-human perforin antibody (clone B-D48, 1:1,000 dilution in 1% BSA in TBST, RRID:AB_2616860). After three washes in TBST, membranes were incubated with Alexa Fluor 790 goat anti-mouse IgG (H + L; Invitrogen, cat. #A11357) at a 1:10,000 dilution for 30 minutes. Finally, membranes were washed in TBST and scanned at 790 nm using the LI-COR system to detect perforin.

### Human CAR T-cell manufacturing

Human CARs were generated by fusing gene block fragments (custom ordered from IDT) into a lentiviral vector. The CAR sequence is composed of a human CD8 signal peptide, scFv, human CD8a hinge and transmembrane domain, CD28 costimulatory domain, and CD3ζ intracellular domain. FMC63 (RRID:AB_94155), Leu16 (B-Ly1, RRID:AB_626969), and m971-long antibody clones were used for CD19, CD20, and CD22 CARs, respectively. To facilitate CAR detection by flow cytometry, a FLAG tag (DYKDDDDK) was inserted at the N-terminus of the scFv immediately following the signal peptide in all three constructs. CD3^+^ T cells were isolated by negative selection using RosetteSep Kits from STEMCELL Technologies and obtained from the Human Immunology Core at the Perelman School of Medicine at the University of Pennsylvania. T cells were activated with Human T-Activator CD3/CD28 Dynabeads (Thermo Fisher Scientific) at a bead-to-cell ratio of 1:1 in complete medium supplemented with 100 IU/mL recombinant human IL2 (PeproTech). After 24 hours of activation, T cells were transduced with lentiviral supernatant containing 5 μg/mL polybrene, and spinfection was performed at 2,000 × *g* for 90 minutes at 35°C. Plates were then carefully transferred to an incubator and maintained overnight. The next day, plates were centrifuged at 600 × *g* for 5 minutes, and the lentivirus-containing supernatant was removed and replaced with new virus. Spinfection was repeated as previously described. Cells were transferred to the incubator, and after 8 hours, the cell media were replenished with new complete media containing 100 IU/mL recombinant human IL2. Transduction efficiencies were determined by flow cytometry 2 days later using an anti-FLAG antibody.

### Statistical analysis

Data are shown as means ± SD or means ± SEM, as indicated in the figure legends. GraphPad Prism 9.5.1 (GraphPad Software; RRID:SCR_002798) was used for statistical analyses, as previously described (19). Data distribution was tested using the Shapiro–Wilk test, D’Agostino–Pearson test, and Kolmogorov–Smirnov test. Statistical analyses of three or more groups were compared using one- or two-way ANOVA or Brown–Forsythe ANOVA, followed by Tukey, Dunnett, or Bonferroni multiple comparisons test, or Kruskal–Wallis test followed by Dunn multiple comparisons test. Repeated measures ANOVA with Šídák or Holm–Šídák *post hoc* tests was used to analyze the differences in paired samples. Statistical analyses of two groups were compared using an unpaired *t* test, a paired *t* test, or the Mann–Whitney test. Methods of statistical analyses are defined in every figure legend. A *P* value of less than 0.05 was considered statistically significant. Each experiment was performed in technical duplicates or triplicates. The number of biological replicates for each experiment is mentioned in the figure legends.

### Data availability

The data analyzed in this study were obtained from www.immunomics.ch/hcc/ (20). The data generated in this study are available from the corresponding author upon request.

## Results

### Cancer-conditioned medium suppresses NK cells

**Figure 1.**
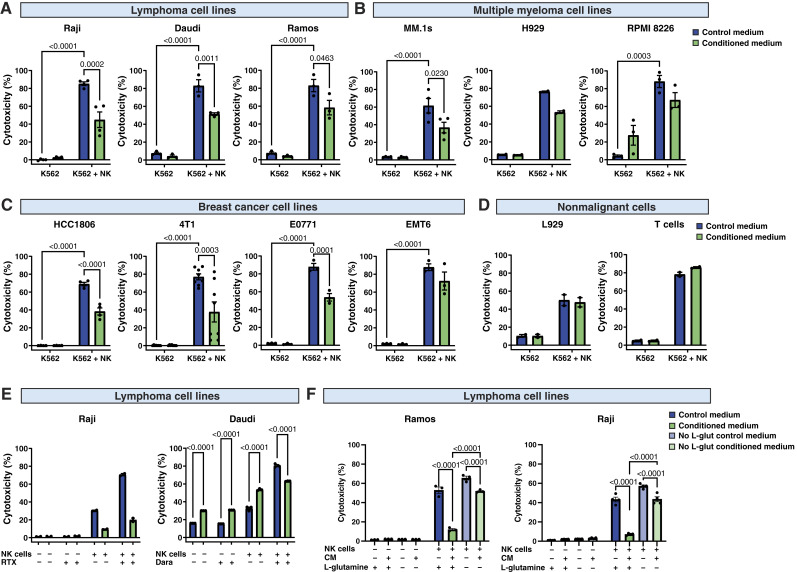
Cancer cell–conditioned medium inhibits natural cytotoxicity and ADCC of NK cells. **A–D,** Natural cytotoxicity of NK cells against K562 cells in the presence of control medium and lymphoma cell conditioned medium (Raji, *n* = 4; Daudi, *n* = 3; Ramos, *n* = 3; **A**), multiple myeloma cells–conditioned medium (MM.1s, *n* = 4; H929, *n* = 2; RPMI 8226, *n* = 3; **B**), breast cancer cell conditioned medium (HCC1806, *n* = 4; 4T1, *n* = 8; E0771, *n* = 3; EMT6, n = 3; **C**), and nonmalignant cell conditioned medium (L929, *n* = 2; T cells, *n* = 2; **D**). For **A–D**, K562 cells were stained with CFSE and incubated with NK cells in medium conditioned by the indicated cells. Cytotoxicity was assessed after 4 hours using flow cytometry as a percentage of propidium iodide–positive CFSE-positive (K562) cells. **E,** ADCC of NK cells against Raji cells with anti-CD20 antibody (100 μg/mL of RTX) and Daudi cells with anti-CD38 antibody (1 μg/mL of daratumumab, Dara) in the presence of control medium or lymphoma cell conditioned medium (*n* = 2). Cytotoxicity was assessed after 4 hours using flow cytometry as a percentage of propidium iodide–positive CFSE-positive cells. **F,** Natural cytotoxicity of NK cells against K562 cells in the presence of control medium or lymphoma cell conditioned medium (Ramos, *n* = 3; Raji, *n* = 4). The normal medium or medium lacking L-glutamine was used as control medium to generate a medium conditioned by cancer. *P* values were calculated using two-way ANOVA with Tukey *post hoc* test. Data show individual values and means ± SEM. *n* values are the numbers of biological replicates in *in vitro* experiments.

To determine the impact of cancer-secreted factors on NK cell activity, we cultured different cell lines for 48 hours to generate cancer-conditioned medium. Then, the natural cytotoxicity of NK cells was determined by their coculture with K562 cells in the undiluted conditioned or control medium. We found that medium conditioned by various tumor cells, such as lymphoma cell lines (Raji, Daudi, and Ramos cells; Fig. 1A), multiple myeloma cell lines (MM.1s, H929; Fig. 1B), and breast cancer cell lines (human HCC1806, murine 4T1, E0771, EMT6; Fig. 1C), significantly inhibited the natural cytotoxicity of NK cells. In contrast, medium conditioned by a nonmalignant fibroblast cell line (L929) and activated human T cells had no effect on the cytotoxicity of NK cells (Fig. 1D).

NK cells may be engaged by monoclonal antibodies to mediate antitumor responses (21). Thus, we subsequently determined whether the cancer-conditioned medium affected the ADCC of NK cells. For this purpose, we employed lymphoma and multiple myeloma cell line models expressing CD20 and/or CD38, respectively, as targets for clinically used antibodies. We found that tumor cell–conditioned medium suppressed RTX- and daratumumab-dependent cell-mediated cytotoxicity of NK cells against CD20^+^ Raji cells and CD38^+^ Daudi cells, respectively (Fig. 1E). Further, to recapitulate the natural architecture of solid tumors more reliably, including the heterogeneous distribution of oxygen and nutrients that differently affect cell metabolism, we utilized a three-dimensional cell culture of both murine (4T1) and human (MDA-MB 231, HCC1806) breast cancer cells (Supplementary Fig. S1A) and found that conditioned medium potently inhibited the cytotoxicity of NK cells (Supplementary Fig. S1B).

To characterize the factors responsible for the suppression of NK cells, we fractionated a representative cancer-conditioned medium from Raji cells using ultrafiltration into two fractions that contained either factors smaller than 3 kDa (predominantly low-molecular-weight metabolites) or larger molecules (predominantly proteins; Supplementary Fig. S2A). It was revealed that only the low-molecular-weight fraction of Raji cell conditioned medium impaired the cytotoxicity of NK cells against target cells (Supplementary Fig. S2B).

### Ammonia accumulates in cancer cell–conditioned medium and TME

**Figure 2.**
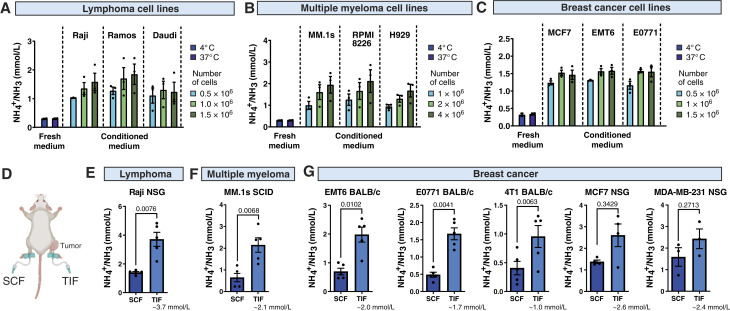
Ammonia concentration is increased in cancer cell–conditioned medium and TIF. Ammonia concentration in the tumor-conditioned medium, collected after 48 hours of incubation, was measured using a Dimension Ammonia assay (Siemens; *n* = 3). Cells were cultured at different densities: lymphoma (0.5 × 10^6^, 1.0 × 10^6^, 1.5 × 10^6^/mL; **A**), multiple myeloma (1 × 10^6^, 2 × 10^6^, 4 × 10^6^/mL; **B**), and breast cancer cell lines (0.5 × 10^6^, 1 × 10^6^, 1.5 × 10^6^/mL; **C**). In **A–C**, empty medium incubated for 48 hours (without the cells) at 4°C or 37°C is presented as a control. **D,** Schematic presentation of the TIF and SCF isolation from mice. TIF was collected from tumors not exceeding 1,500 mm^3^. SCF was isolated at the same time from the contralateral tight as a control tissue fluid. **E–G,** The concentration of ammonia in TIF and SCF isolated from Raji tumor–bearing NSG (*n* = 5; **E**), MM.1s tumor–bearing SCID mice (*n* = 5; **F**), and breast cancer–bearing mice (EMT6 in BALB/c mice, *n* = 5; E0771 in BALB/c mice, *n* = 5; 4T1 in BALB/c mice; *n* = 5; MCF7 in NSG mice, *n* = 4; MDA-MB-231 in NSG mice, *n* = 3; **G**). *P* values were calculated using a paired *t* test. Data show individual values and means ± SEM. *n* values are the number of biological replicates in *in vitro* experiments or the number of mice used to obtain the data. **D,** Created with BioRender.com. Winiarska, M. (2025) https://BioRender.com/x56a982.

Based on the literature that demonstrated the accumulation of ammonia in TME and its inhibitory effects on different populations of immune cells, we hypothesized that it may contribute to the inhibitory effects of the low-molecular-weight fraction of cancer-conditioned medium on NK cell activity. To validate this hypothesis, we generated conditioned media in the absence of L-glutamine, a main source of ammonia production by tumor cells (22). We observed that conditioned media without glutamine still inhibited the cytotoxicity of NK cells but significantly less than regular medium with L-glutamine (Fig. 1F). Therefore, we further measured ammonia secretion by different cancer cell lines. Accumulation of ammonia was detected in media conditioned by all investigated tumor cell lines, including lymphoma (Fig. 2A), multiple myeloma (Fig. 2B), and breast cancer cell lines (Fig. 2C), as compared with control media stored at 4°C or 37°C. Notably, conditioning of the media by cancer cells only slightly decreased the pH of the collected supernatants (Supplementary Fig. S3A). To determine whether ammonia accumulation also occurs *in vivo*, we isolated TIF that represents TME from different types of tumors in mice and measured ammonia concentration in comparison with SCF (Fig. 2D). In these experiments, both immunocompetent BALB/c mice and immunocompromised SCID or NSG mice were used to inoculate murine and human cell lines, respectively. In both syngeneic and xenograft models, we found that ammonia accumulated in the TME in all types of studied tumor models, including lymphoma (Fig. 2E), multiple myeloma (Fig. 2F), and breast cancer tumors (Fig. 2G). The concentrations of ammonia in TIFs varied between different tumors from 0.5 to 5 mmol/L, which remains consistent with already published data (11), and were, in general, even higher than those measured in cancer-conditioned medium *in vitro*. Notably, the production of ammonia by tumor cells *in vitro* was completely blocked in a glutamine-free medium (Supplementary Fig. S3B). These results, along with the lack of an inhibitory effect of glutamine-free conditioned medium, suggest that ammonia is involved in inhibiting NK cell cytotoxicity.

### Ammonia suppresses NK cells

**Figure 3.**
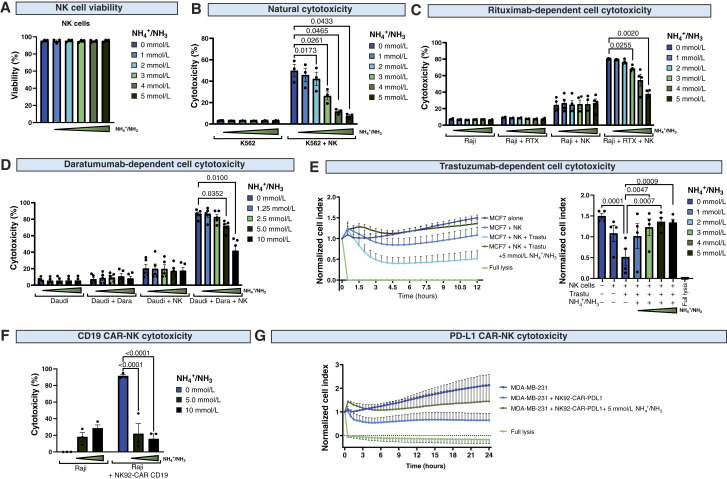
Ammonia inhibits natural cytotoxicity and ADCC of NK cells and CAR NK cells. **A,** The viability of NK cells incubated with different concentrations of NH_4_Cl for 4 hours was assessed using propidium iodide staining and flow cytometry (*n* = 3). **B,** Natural cytotoxicity of NK cells against K562 cells in the presence of different concentrations of NH_4_Cl (*n* = 3). K562 cells were stained with CFSE and incubated with NK cells in different concentrations of ammonia. **C,** RTX-dependent cell cytotoxicity of NK cells against Raji cells in the presence of different concentrations of NH_4_Cl (*n* = 4). Raji cells were stained with CFSE and incubated with NK cells and 100 μg/mL RTX in different concentrations of ammonia. **D,** Daratumumab (Dara)-dependent cell cytotoxicity of NK cells against Daudi cells in the presence of different concentrations of NH_4_Cl (*n* = 5). Daudi cells were stained with CFSE and incubated with NK cells and 1 μg/mL daratumumab in different concentrations of ammonia. In **B–D**, cytotoxicity was assessed after 4 hours using flow cytometry and plotted as the percentage of propidium iodide–positive CFSE-positive target tumor cells. **E,** Trastuzumab-dependent cell cytotoxicity of NK cells against MCF7 cells in the presence of different concentrations of NH_4_Cl (*n* = 4). Cytotoxicity was assessed using RTCA for 12 hours. The right panel presents the normalized cell index at the 12-hour time point. **F,** CD19 CAR NK cell cytotoxicity against Raji cells in the presence of different concentrations of NH_4_Cl (*n* = 3). Cytotoxicity was determined after 18 hours in a luciferase-based killing assay, with Raji cells stably expressing luciferase as target cells. **G,** PD-L1 CAR NK cell cytotoxicity against MDA-MB-231 cells in the presence of 5 mmol/L NH_4_Cl (*n* = 3). Cytotoxicity was assessed using RTCA for 24 hours. *P* values were calculated using two-way ANOVA with Tukey *post hoc* test. Data show individual values and means ± SEM. *n* values are the numbers of biological replicates in *in vitro* experiments.

To determine whether ammonia regulates the activity of NK cells, we tested the influence of exogenously added ammonia in a range of concentrations corresponding to those observed *in vivo* in TIF. Ammonia did not affect the viability of NK cells (Fig. 3A), even in long-term settings (Supplementary Fig. S4A). Nonetheless, we found that ammonia accumulated within NK cells (Supplementary Fig. S4B), similarly to what was demonstrated in T cells (16).

Notably, ammonia inhibited the natural cytotoxicity of NK cells in a dose-dependent manner (Fig. 3B). At concentrations observed in most cancer-conditioned media and TIFs (2–3 mmol/L), ammonia substantially suppressed NK cell cytotoxicity, and at higher concentrations that were still within a range of *in vivo* measurements (4–5 mmol/L), it completely inhibited NK cell killing activity. Moreover, the suppression of NK cell cytotoxicity was even more pronounced when NK cells were preincubated with ammonia for up to 48 hours before contact with target cells (Supplementary Fig. S4C).

Using RTX and daratumumab, we demonstrated that ammonia-suppressed ADCC against Raji (Fig. 3C) and Daudi cells (Fig. 3D), reproducing the effects of a cancer-conditioned medium (Fig. 1E). Moreover, ammonia inhibited trastuzumab-dependent ADCC against breast cancer cells, as assessed by the RTCA assay (Fig. 3E). In addition to the engagement of NK cells by monoclonal antibodies, NK cells can be redirected to the tumor cells by the introduction of tumor-targeting CARs. Using the NK-92 cell line transduced with CD19 (FMC63 scFv) or PD-L1 (atezolizumab-based scFv) CARs, we found that ammonia suppressed the cytotoxicity of NK-92 CAR cells against Raji (Fig. 3F) and MDA-MB-231 breast cancer cells (Fig. 3G), respectively. Altogether, these data demonstrate that ammonia potently suppresses the antitumor activity of NK cells and NK cell–based therapeutic strategies.

To further determine the effects of ammonia on the antitumor activity of NK cells *in vivo*, we used BALB/c SCID mice characterized by an absence of functional T cells and B cells that were inoculated subcutaneously with Raji cells. Mice were treated systemically with RTX, and 50 mmol/L of NH_4_Cl was administered intratumorally (Supplementary Fig. S5A). Mice that were injected intratumorally with ammonia and RTX exhibited a trend toward shorter survival (Supplementary Fig. S5B) and greater tumor volume (Supplementary Fig. S5C) compared with the control mice treated with RTX only. Similar results were obtained when RTX and NH_4_Cl were administered for a longer duration (Supplementary Fig. S5D and S5E). According to the previous study (11), ammonia administered systemically as a bolus of 9 mmol/kg NH_4_Cl is rapidly recycled in the TME into amino acids by cancer cells. We found that after local administration of 50 mmol/L of NH_4_Cl directly into tumor masses (Supplementary Fig. S6A), the ammonia was rapidly utilized or excreted from the TME because no significant changes in the ammonia concentration were detected in the TIFs of NH_4_Cl-injected tumors after 0.5 or 4 hours (Supplementary Fig. S6B). Finally, we found that even the administration of a supraphysiologic dose of NH_4_Cl (100 mmol/L) into the TME did not change the ammonia concentration in the TIFs when measured 30 minutes after administration (Supplementary Fig. S6C). Nonetheless, based on *in vitro* data and *ex vivo* ammonia measurements in TIF isolated from tumor masses, we sought to determine the mechanism of the suppressive effects of ammonia on NK cell activity.

### Ammonia decreases the level of perforin in NK cells

**Figure 4.**
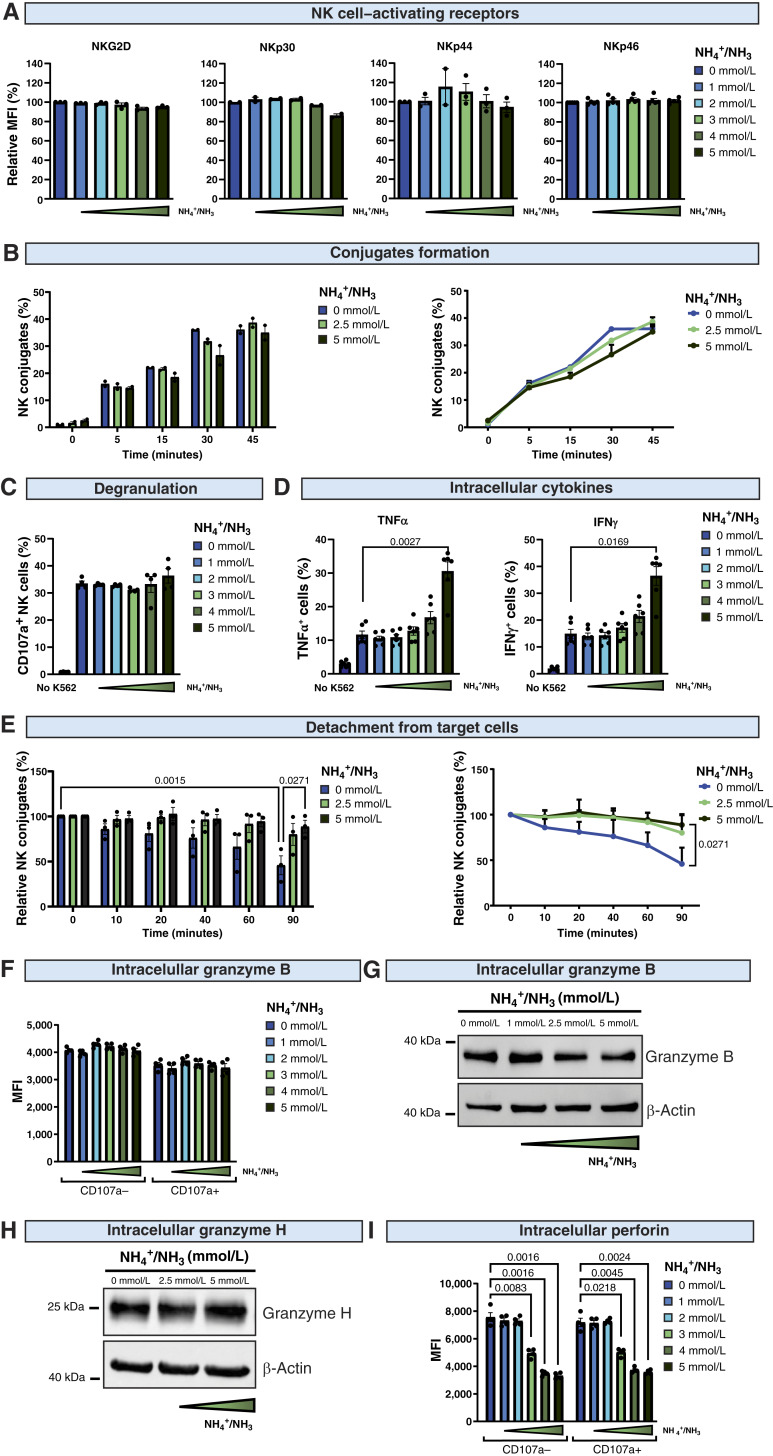
Ammonia affects NK cell functions and decreases the level of perforin. **A,** The surface levels of NK cell activation receptors (NKG2D, NKp30, NKp44, NKp46) after incubation with different concentrations of NH_4_Cl were assessed after 4 hours using flow cytometry. Mean fluorescence intensity (MFI) was normalized to control (medium without exogenously added ammonium). **B,** Formation of conjugates of NK and target cells in different concentrations of NH_4_Cl (*n* = 3). NK cells were stained with CellTrace Deep Red (CTDR), and target cells (K562 cells) were stained with CFSE. Cells were mixed and incubated with different concentrations of ammonia at 37°C for 30 minutes. After this time, 4% PFA was added, followed by a 10-minute incubation and wash. The percentage of conjugates defined as CTDR^+^CFSE^+^ double-positive events was determined using flow cytometry. The graph shows a representative experiment from one donor. **C,** Degranulation of NK cells in response to stimulation with target cells (K562) at different concentrations of NH_4_Cl (*n* = 4). The percentage of degranulating NK cells (CD107a^+^) was assessed after 4 hours using flow cytometry. **D,** The level of cytokines expressed in NK cells in response to stimulation with target cells (K562) at different concentrations of NH_4_Cl (*n* = 6). The percentage of TNFα-positive and IFNγ-positive NK cells was assessed after 4 hours using flow cytometry. **E,** Detachment of NK cells from target cells in different concentrations of NH_4_Cl (*n* = 3). NK cells were stained with CTDR, and target cells (K562 cells) were stained with CFSE. Cells were mixed and incubated in different concentrations of ammonia. After 0 to 45 minutes, 4% PFA was added, followed by a 10-minute incubation and wash. The percentage of conjugates, defined as CTDR^+^CFSE^+^ double-positive events, was determined using flow cytometry. **F,** The level of granzyme B in NK cells incubated in different concentrations of NH_4_Cl (*n* = 4). The level of granzyme B in CD107a^−^ and CD107a^+^ NK cells was assessed by intracellular staining using flow cytometry. **G,** The level of granzyme B in NK cells incubated with ammonia determined by Western blot methods using an anti–granzyme B antibody (M3304B06 clone; *n* = 2). β-Actin is presented as a loading control. Representative blot from one donor is shown. **H,** The level of granzyme H in NK cells incubated with ammonia determined by Western blot methods using an anti–granzyme H antibody (E3H7W clone; *n* = 2). β-Actin is presented as a loading control. Representative blot from one donor is shown. **I,** The level of perforin in NK cells incubated in different concentrations of NH_4_Cl (*n* = 4). The level of total perforin in CD107a^−^ and CD107a^+^ NK cells was assessed by intracellular staining (B-D48 antibody) using flow cytometry. *P* values were calculated using two-way ANOVA with Tukey post hoc test. Data show means ± SEM. *n* values are the numbers of biological replicates in *in vitro* experiments.

The process of killing target cells by NK cells consists of several steps, which include NK cell activation, conjugation with target cells, degranulation, cytokine production, and detachment from target cells (23). We found that ammonia did not affect the surface levels of NK cell–activating receptors NKG2D, NKp30, NKp44, and NKp46 (Fig. 4A). Moreover, there was no impairment in the formation of conjugates of NK and target cells in the presence of ammonia (Fig. 4B). After the formation of conjugates, ammonia did not influence the percentage of degranulating NK cells (Fig. 4C). Simultaneously, high ammonia concentration increased the percentage of TNFα/IFNγ-expressing NK cells (Fig. 4D). Moreover, we observed delayed detachment of NK cells from target cells at high ammonia concentrations (Fig. 4E). These findings suggested that in the presence of ammonia, NK cells are still fully functional in attaching to target cells and releasing the content of secretory lysosomes (degranulating). Nonetheless, they are unable to execute target cell killing, which is a signal for disengagement from target cells (24). Therefore, we determined the levels of granzyme B, granzyme H, and perforin, which are major cytolytic proteins within secretory lysosomes (25). Although the levels of granzyme B (Fig. 4F and G) and granzyme H (Fig. 4H) were unaffected even in high concentrations of ammonia, we found that the level of perforin was significantly decreased by ammonia in a dose-dependent manner when measured by flow cytometry (Fig. 4I).

### Ammonia decreases the amount of mature perforin

**Figure 5.**
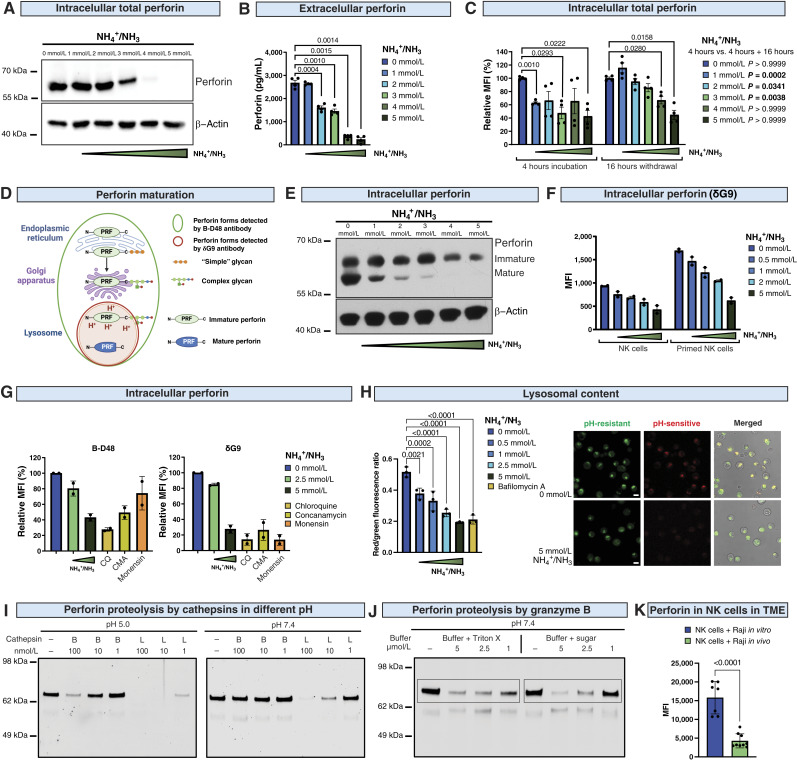
Ammonia decreases the amount of mature perforin in NK cells. **A,** The level of total perforin in NK cells incubated with ammonia determined by Western blot using an anti-perforin antibody (B-D48 clone; *n* = 3). β-Actin is presented as a loading control. Representative blot from one donor is shown. **B,** The concentration of extracellular perforin secreted by NK cells in response to contact with target cells (K562) in different concentrations of NH_4_Cl (*n* = 4). **C,** The level of perforin detected in NK cells incubated with NH_4_Cl determined by intracellular staining using an anti-perforin antibody (δG9 clone) and flow cytometry (*n* = 4). In some groups, cells were washed and incubated in a medium without NH_4_Cl, as indicated in the figure. MFI, mean fluorescence intensity. **D,** A schematic representation of perforin maturation. Green and red circles show forms of perforin recognized by B-D48 and δG9 antibodies, respectively. **E,** The level of perforin in NK cells incubated for 4 hours with NH_4_Cl determined by Western blot methods using an anti-perforin antibody (Pf-344 clone; *n* = 3). Two forms of perforin, immature (70 kDa) and mature (60 kDa), were detected. **F,** The level of perforin detected in NK cells incubated with NH_4_Cl determined by intracellular staining using an anti-perforin antibody (δG9 clone) and flow cytometry (*n* = 2). NK cells were primed with IL2 (200 U/mL) and IL15 (10 ng/mL) for 24 hours before the experiment. **G,** The level of perforin detected in NK cells incubated with NH_4_Cl and other lysosomotropic agents [chloroquine (CQ), concanamycin (CMA), and monensin] for 4 hours determined by intracellular staining using antibodies detecting total perforin (B-D48 clone) and lysosomal perforin (δG9 clone; *n* = 2). **H,** The cells were loaded with the lysosomal fluorescent probes LysoPrime Green (the pH-resistant probe) and pHLys Red (the pH-sensitive probe; Dojindo Laboratories) and treated with increasing concentrations of NH_4_Cl in the imaging medium or bafilomycin A as a control. Bars show calculated mean ratios of the LysoPrime Green signal divided by the pHLys Red signal (*n* = 3). Images show cells that were untreated (top row) and treated with 5 mmol/L NH_4_Cl (bottom row). Left column, staining with the pH-resistant LysoPrime Green probe (green; scale bar, 10 μm); middle column, staining with the pH-sensitive pHLys Red probe (red); right column, merge of the LysoPrime Green signal, pHLys Red signal, and transmitted light image (gray). **I,** pH-dependent processing of perforin by human recombinant cathepsins B and L at concentrations ranging from 1 to 100 nmol/L, analyzed by Western blot methods using an anti-perforin antibody (B-D48 clone; *n* = 2). **J,** Processing of perforin by human recombinant granzyme B at concentrations of 0.5, 1, and 2.5 μmol/L, assessed at pH 7.4 in two different buffers (*n* = 2). **K,** Raji tumor–bearing mice were intratumorally injected with 3–5 × 10^6^ human NK cells. After 4 hours, tumors were dissected and enzymatically dissociated, followed by NK cell analysis for perforin levels using intracellular staining with an anti-perforin antibody (δG9 clone) and flow cytometry (*n* = 9). Human NK cells incubated with Raji cells for 4 hours in control medium *in vitro* were used as controls. Data show means ± SEM. The *n* values represent the numbers of biological replicates in *in vitro* experiments or the number of mice used to obtain the data. **D,** Created with BioRender.com. Winiarska, M. (2025) https://BioRender.com/g06z848.

We further confirmed that ammonia reduces perforin levels in NK cells using Western blot (Fig. 5A). A similar downregulation of perforin was observed when NK cells were incubated in a cancer-conditioned medium (Supplementary Fig. S7A). Notably, ammonia not only decreased intracellular perforin in NK cells but also decreased the total amount of perforin that was secreted by the NK cells in response to cancer cell recognition (Fig. 5B). We observed a similar trend toward decreased extracellular perforin levels when NK cells were incubated with target cells in cancer-conditioned media (Supplementary Fig. S7B–S7D). Notably, we observed a reversible downregulation of perforin by low doses of ammonia, as after 16 hours of ammonia washout, the amount of perforin returned to control levels (Fig. 5C). In addition, ammonia had no effect on perforin at the transcriptional level (Supplementary Fig. S8). These findings suggest that the decrease in perforin upon contact with ammonia is a relatively fast and transcription-independent process, most probably related to its maturation and/or degradation.

Perforin is synthesized as a 70 kDa inactive precursor that undergoes several post-translational modifications, including proteolytic cleavage in acidic compartments. This process yields a 60 kDa mature perforin, which is associated with proteoglycans and stored in the lysosomes until secretion in response to stimuli (Fig. 5D). Thus, it is possible to distinguish immature and mature perforin based on their molecular size (26–28). Indeed, we detected both forms of perforin in primary NK cells by Western blotting using the Pf-344 antibody, which binds a linear epitope within the EGF-like domain (29). Notably, ammonia decreased the mature perforin level at a concentration as low as 1 mmol/L (Fig. 5E). Mature perforin completely disappeared in high (4–5 mmol/L) NH_4_Cl concentrations. It is worth noting that the decrease in mature perforin was not accompanied by an increase in the immature form. Further, we confirmed by flow cytometry the reduction of perforin amount using the δG9 antibody (Fig. 5F) that recognizes the pH-sensitive motif in granule-associated perforin (Fig. 5D; ref. 30). Perforin detected by the δG9 antibody was decreased in NK cells treated with ammonia, which was even more pronounced in NK cells primed with IL2 and IL15 that potently expressed perforin (Fig. 5F).

An essential role in the storage and maturation of perforin within the lytic granules is played by an acidic pH, which provides a general mechanism for NK cells to keep their lytic machinery functional (Fig. 5D; ref. 31). As it was reported that ammonia is a lysosomotropic agent (32), we hypothesized that the decreased amount of perforin may be caused by an increased pH in lysosomes. We found that other lysosomotropic agents, including chloroquine, concanamycin, and monensin, exerted effects on perforin similar to those of ammonia, with the most pronounced changes in mature perforin detected by the δG9 antibody (Fig. 5G). To determine whether changes in lysosomes mediate the effects of ammonia on perforin, we utilized a LysoTracker probe, which selectively accumulates in acidic organelles. Indeed, we demonstrated that ammonia decreased the acidic compartment in NK cells (Supplementary Fig. S9). To further investigate pH changes in acidic organelles, we utilized lysosomal dyes, pHLys Red (pH-sensitive) and LysoPrime Green (pH-resistant), which enable the detection of both lysosomal pH and lysosomal mass. These experiments confirmed that ammonia increases pH in acidic organelles to a level similar to that of bafilomycin A (33), an inhibitor of vacuolar-type ATPase (V-ATPase)-dependent lysosomal acidification (Fig. 5H; Supplementary Fig. S10). Then, we analyzed CD63 (LAMP3) and CD107a (LAMP1), markers of secretory lysosomes (34), in NK cells incubated with different doses of NH_4_Cl (Supplementary Fig. S11A). We observed that ammonia increased the number of LAMP1^+^ vesicles (Supplementary Fig. S11B) but decreased their area (Supplementary Fig. S11C) and mean LAMP1 level in NK cells (Supplementary Fig. S11D). Ammonia had no effect on CD63^+^ vesicles. Altogether, these observations suggest that ammonia selectively upregulates pH in acidic organelles.

Furthermore, to better understand the fate of perforin upon pH increase, we performed experiments in which NK cells were treated with cycloheximide (CHX), a compound that blocks protein translation. Immature perforin decreased already 2 hours after CHX treatment (Supplementary Fig. S12). However, within 4 hours (the same time frame in which we observed the decrease of mature perforin upon ammonia treatment), the amount of mature perforin was stable and not affected by CHX treatment (Supplementary Fig. S12). These results suggest that ammonia rather promotes perforin degradation than impairs its maturation.

To identify potential degrading hydrolases, we performed enzymatic cleavage studies in cell-free assays with recombinant mature perforin. In these experiments, we analyzed the degradation of perforin by cathepsin B, cathepsin L, and granzyme B (Fig. 5I and J) after 30 minutes of incubation at pH 5.0 and pH 7.4. We observed that both cathepsin L and granzyme B degrade recombinant mature perforin (Fig. 5I and J). Although cathepsin L has already been reported by others to be involved in the maturation of perforin in T/NK cells (35), we hypothesize that this particular cathepsin can also be involved in perforin degradation. Although cathepsin L is active at acidic pH (27, 35–37), it can also retain some of its activity at higher pH, as reported by others (38, 39) and also observed in our cell-free experiments (Fig. 5I, right panel). In particular, cathepsin L was able to degrade perforin at pH 7.4; however, the efficacy of degradation was evidently lower compared with pH 5.0. At pH 7.4, optimal for granzyme B, we also detected dose-dependent degradation of perforin by granzyme B. These data suggest that after alkalization of the lysosome and perforin dissociation from proteoglycans, cathepsins and granzyme B can lead to its proteolysis.

Further, to validate our findings in *in vivo* settings, we intratumorally injected Raji tumor–bearing mice with human NK cells, and after 4 hours (the same time as *in vitro* experiments), we dissected tumors and analyzed lysosomal perforin in NK cells within the TME. After this time in the TME of Raji tumors, in which we observed a ∼3.7 mmol/L concentration of ammonia, the levels of perforin were significantly decreased in NK cells when compared with NK cells cocultured with Raji cells in control medium *in vitro* (Fig. 5K). Collectively, we demonstrated that mature perforin levels are decreased in NK cells both *in vitro* and *in vivo* and that this effect is dependent on pH changes in the acidic compartment of NK cells.

### Ammonia inhibits NK cell serial killing

**Figure 6.**
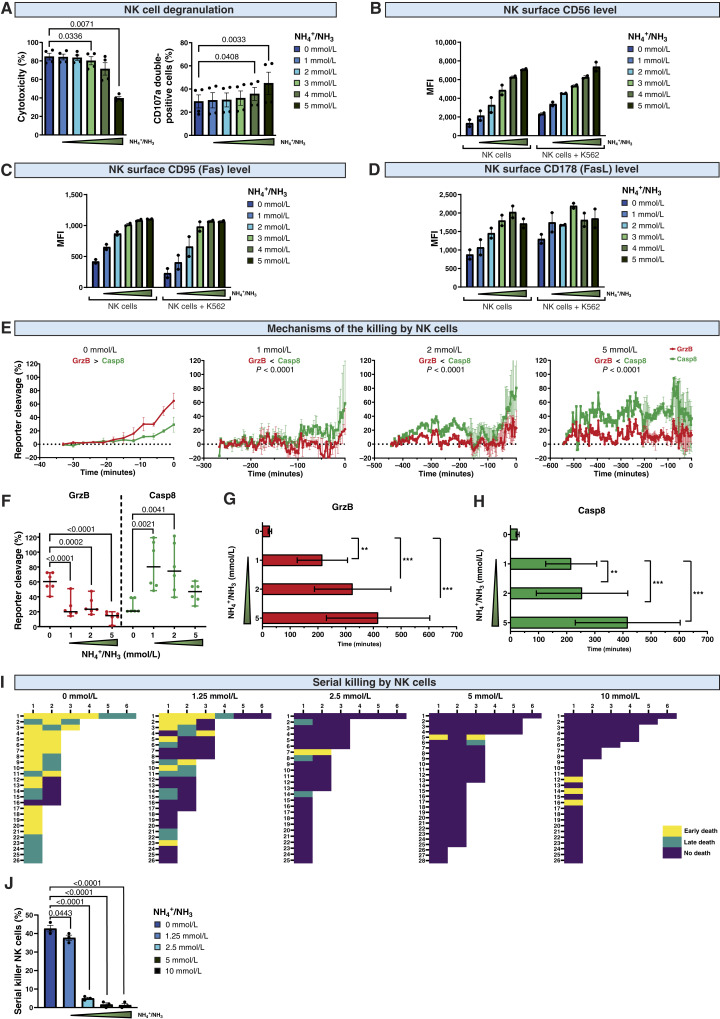
Ammonia inhibits NK cell serial killing. **A,** The percentage of NK cells that underwent serial degranulation in response to stimulation with target cells (K562) in different concentrations of NH_4_Cl (*n* = 4). Cells were stained with anti-CD107a after 2 hours of incubation, and then after an additional 2 hours, they were stained with an anti-CD107a antibody conjugated with a different fluorochrome and analyzed using flow cytometry. NK cells that underwent serial degranulation were defined as CD107a-double positive NK cells. **B,** The level of CD56 in NK cells incubated with NH_4_Cl, with or without target cells, determined by surface staining and flow cytometry (*n* = 2). **C,** The level of CD95 (Fas) in NK cells incubated with NH_4_Cl with or without target cells determined by surface staining and flow cytometry (*n* = 2). **D,** The level of CD178 (FasL) in NK cells incubated with NH_4_Cl with or without target cells determined by surface staining and flow cytometry (*n* = 2). **E–K,** HeLa cells were transfected with NES-ELQTD-GFP-T2A-NES-VGPD-mCherry and CD48. In these cells, NES-RIEADS-mCherry (activation of GrzB) and NES-VGPD-mGFP [activation of caspase-8 (Casp8)] reporter cleavage can be detected by the appearance of fluorescence inside the nucleus. GrzB cleaves the reporter, resulting in an increase in the red fluorescent signal in the nucleus, whereas the caspase-8 reporter is specifically activated by death receptor–mediated apoptosis and results in an increase of green fluorescence in the nucleus. Confocal time-lapse microscopy started immediately after NK cell exposure. **E,** Activation of granzyme B (GrzB) and caspase-8 reporters in target HeLa cells incubated with NK cells in different concentrations of NH_4_Cl. Time point 0 corresponds to the time of cell death (*n* = 15). Images were acquired every 3 minutes. **F,** Activation of granzyme B and caspase-8 reporters in target HeLa cells incubated with NK cells in different concentrations of NH_4_Cl at the time of cell death (*n* = 6). **G,** Time required to kill a reporter-expressing cell by NK cells in different concentrations of NH_4_Cl through the activation of granzyme B (*n* = 15). SYTOX Blue dye was used to assess the viability of the target cell. Early or late cell death was evaluated based on the kinetics of the target cell nucleus staining after contact with NK cells. **H,** Time required to kill a reporter-expressing cell by NK cells in different concentrations of NH_4_Cl by activation of caspase-8 (*n* = 15). **I,** The diagram displays target cell death in single NK cell–target cell contacts. Each row displays the contact sequence of one individual NK cell imaged using time-lapse microscopy (controls, *n* = 26; 1.25 mmol/L, *n* = 26; 2.5 mmol/L, *n* = 25; 5 mmol/L, *n* = 28; 10 mmol/L, *n* = 27). **J,** Percentage of serial killers among NK cells in different concentrations of NH_4_Cl (controls, *n* = 26; 1.25 mmol/L, *n* = 26; 2.5 mmol/L, *n* = 26; 5 mmol/L, *n* = 28; 10 mmol/L, *n* = 27). Label-free count (LFC) was obtained from label-free quantification. *P* values were calculated using two-way ANOVA with Tukey post hoc test. Data show means ± SEM. *n* values are the numbers of biological replicates in *in vitro* experiments. MFI, mean fluorescence intensity.

Finally, we sought to determine the consequences of ammonia-impaired NK cell effector functions. Decreased cytotoxicity of NK cells was associated with an increased percentage of NK cells that degranulated more than once (serial degranulation; Fig. 6A, right panel), which may be caused by the lack of signals promoting NK cell detachment and a second attempt to kill the target cell. Ammonia increased the membrane expression of CD56 on both resting and target-activated NK cells (Fig. 6B). Moreover, it increased the levels of both Fas (CD95, Fig. 6C) and FasL (CD178, Fig. 6D) on NK cells, suggesting a switch to death receptor–mediated cytotoxicity.

To verify whether ammonia inhibits granzyme-dependent killing and leads to a compensatory upregulation of death receptor–mediated cytotoxicity, we used the HeLa cell line that expresses two reporters enabling quantification of granzyme B and caspase-8 activity in living cells by fluorescence measurement (40). This method allows live tracking of target killing by NK cells and distinguishes between perforin-/granzyme B–mediated death and death receptor–mediated killing (41). Live-cell imaging of control NK cells incubated with reporter-expressing cancer cells revealed that the dominant mechanism of killing depends on perforin/granzyme B. However, in the presence of NH_4_Cl, this mechanism was severely impaired even at low concentrations of NH_4_Cl (Fig. 6E). Ammonia induced a switch from perforin/granzyme B to death receptor–mediated cytotoxicity, as revealed by increased cleavage of the caspase-8 reporter (Fig. 6F). Notably, live-cell imaging revealed that even at low NH_4_Cl concentrations, NK cells needed significantly more time to kill the target cells. For example, in 2 mmol/L of NH_4_Cl, the time from contact with the target cell to successful killing was prolonged by about 10 times, from 27 minutes for granzyme B and 25.5 minutes for caspase-8 to 254.5 minutes and 325.25 minutes, respectively (Fig. 6G and H).

An important feature of NK cells is the ability to detach from the target cell, followed by binding to and killing other cells in a process called serial killing (24). However, this process is largely limited by the availability of lytic granules containing perforin and granzyme B (42, 43). To investigate whether ammonia affects serial killing, we tracked the killing sequence of NK cells incubated with cancer cells in individual microwells. We found that each NK cell was able to kill, on average, 1.5 target cells (Fig. 6I). However, in the presence of ammonia, the average number of cells killed by one NK cell substantially decreased, leading to a profound reduction in the population of NK cells that were serial killers (Fig. 6J). In the presence of ammonia, most of the NK cell–target cancer cell interactions were unsuccessful and did not lead to the death of target cells. These results revealed that ammonia, by impairing perforin-mediated cell killing, substantially prolongs the time needed for NK cells to kill cancer cells, inhibits serial killing, induces a switch toward death receptor–mediated cytotoxicity, and significantly reduces the effectiveness of cancer cell killing by NK cells.

### Ammonia decreases perforin in T cells and inhibits CAR T-cell cytotoxicity

**Figure 7.**
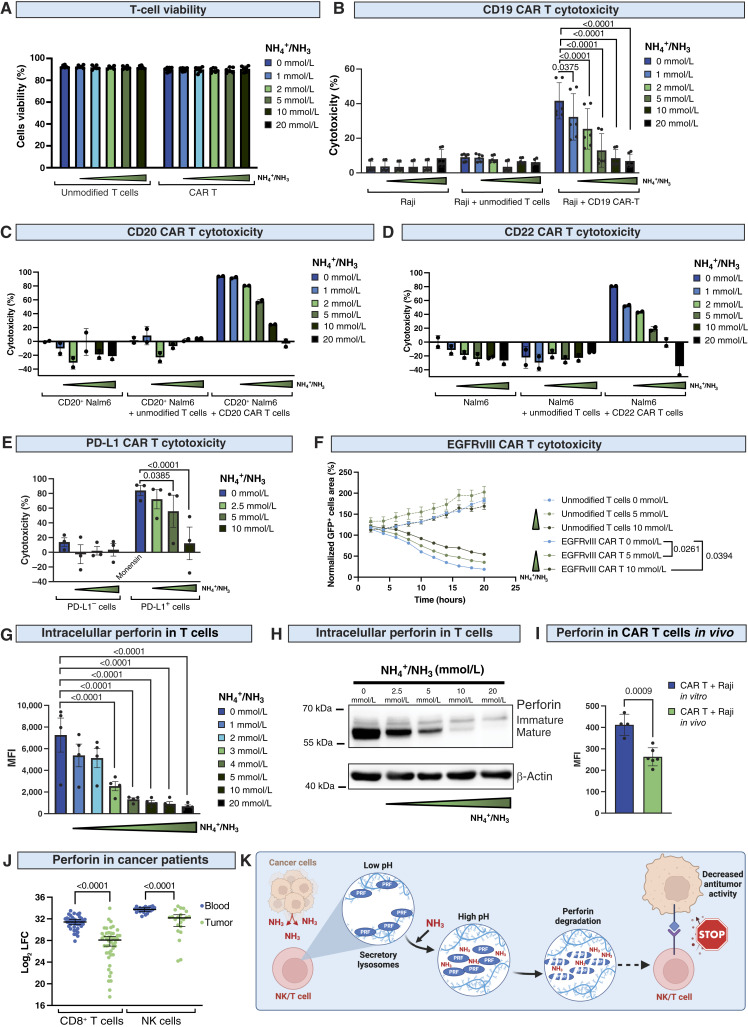
Ammonia decreases perforin levels and inhibits the cytotoxicity of CAR T cells. **A,** Viability of unmodified T cells and CD19 CAR T cells incubated with different concentrations of NH_4_Cl for 6 hours was assessed using propidium iodide staining and flow cytometry. Shown are data from technical replicates (*n* = 2). **B,** CD19 CAR T-cell cytotoxicity against Raji cells in the presence of different concentrations of NH_4_Cl. Cytotoxicity was determined after 6 hours using flow cytometry. Shown are data from technical replicates (*n* = 3). **C,** CD20 CAR T-cell cytotoxicity against luciferase-expressing CD20^+^ Nalm6 cells in the presence of different concentrations of NH_4_Cl. Cytotoxicity was determined after 16 hours and normalized to CD20^+^ Nalm6 cells without CAR T cells. Shown are data from one donor (*n* = 2). **D,** CD22 CAR T-cell cytotoxicity against luciferase-expressing Nalm6 cells in the presence of different concentrations of NH_4_Cl. Cytotoxicity was determined after 16 hours and normalized to Nalm6 cells without CAR T cells. Shown are data from one donor (*n* = 2). **E,** PD-L1 CAR T-cell cytotoxicity against luciferase-expressing PD-L1 knockout or PD-L1^+^ MDA-MB-231 cells in the presence of different concentrations of NH_4_Cl. Cytotoxicity was determined after 16 hours and normalized to MDA-MB-231 cells without CAR T cells (*n* = 3). **F,** EGFRvIII CAR T-cell cytotoxicity against GFP-expressing EGFRvIII^+^ U87 cells in the presence of different concentrations of NH_4_Cl. Cytotoxicity was measured using live-cell microscopy (IncuCyte) for 20 hours. Shown are data from one donor (*n* = 2). **G,** The level of perforin detected in human T cells stimulated with anti-CD3/CD28 and incubated with NH_4_Cl for 4 hours determined by intracellular staining using an anti-perforin antibody (δG9 clone) and flow cytometry (*n* = 4). MFI, mean fluorescence intensity. **H,** The level of total perforin in T cells stimulated with αCD3/CD28 and incubated with ammonia determined by Western blot methods using an anti-perforin antibody (Prf-344 clone). β-Actin is presented as a loading control. Representative blot from one donor is shown. **I,** Raji tumor–bearing mice were intratumorally injected with 5 × 10^6^ human CD19 CAR T cells. After 4 hours, tumors were dissected and enzymatically dissociated, followed by CAR T-cell analysis for perforin levels using intracellular staining with an anti-perforin antibody (δG9 clone) and flow cytometry (*n* = 6). CD19 CAR T cells incubated with Raji cells for 4 hours in control medium *in vitro* were used as controls. **J,** Level of perforin protein in the CD8^+^ T cells and NK cells in the blood and tumors of patients with HCC analyzed from a published dataset (20). **K,** Ammonia increases pH in secretory lysosomes, which results in the dissociation of perforin from proteoglycans. Subsequently, dissociated perforin is susceptible to inactivation and proteolytic degradation. Data show means ± SEM or median ±95% CI for **J**. *n* values are the number of biological replicates in *in vitro* experiments or the number of mice used to obtain the data. **K,** Created with BioRender.com. Winiarska, M. (2025) https://BioRender.com/t34e524.

Cancer immunotherapy utilizes not only NK cells but also T cells (3). Therefore, we further investigated the impact of ammonia on T-cell cytotoxic activity. Similarly to NK cells, ammonia had no impact on T cells or CAR T-cell viability (Fig. 7A; Supplementary Fig. S13A). Further, we assessed the influence of NH_4_Cl on the cytotoxicity of different CAR T-cell products. We found that ammonia significantly inhibited the cytotoxicity of CD19 CAR T cells against Raji cells (Fig. 7B), CD20 CAR T cells against CD20^+^ Nalm6 cells (Fig. 7C), and CD22 CAR T cells against Nalm6 cells (Fig. 7D). Moreover, we demonstrated that ammonia inhibits the cytotoxicity of CAR T cells that are under development for patients with solid tumors. This included PD-L1 CAR T-cell activity against PD-L1^+^ MDA-MB-231 breast cancer cells (Fig. 7E) and EGFR variant III CAR T-cell activity against EGFRvIII^+^ U87 glioblastoma cell line (Fig. 7F). Detailed studies revealed that ammonia decreases the level of perforin in T cells (Fig. 7G). Western blot analysis revealed a loss of mature perforin in both T cells (Fig. 7H) and CD19 CAR T cells (Supplementary Fig. S13B). Similarly to NK cells, ammonia in T cells had no impact on granzyme B levels (Supplementary Fig. S13C) and did not affect perforin at the transcriptional level (Supplementary Fig. S13D).

Further, we intratumorally injected Raji tumor–bearing mice with CD19 CAR T cells and measured the level of perforin by flow cytometry. We observed that after 4 hours in the TME, CD19 CAR T cells had significantly decreased levels of perforin compared with CD19 CAR T cells cocultured with Raji cells in control media *in vitro* (Fig. 7I). To validate these findings in patients with cancer, we analyzed a published proteomics dataset of different immune cell populations in the blood and TME of patients with hepatocellular carcinoma (HCC; ref. 20). These analyses revealed that the amount of perforin at the protein level was significantly decreased in NK cells and T cells infiltrating HCC tumors compared with their counterparts in the blood (Fig. 7J). Notably, in both NK cells (Fig. 5E) and T cells (Fig. 7H), mature perforin is the dominant form and represents more than 90% of total perforin, suggesting that the decreased perforin level can be largely attributed to the loss of mature perforin in tumor-infiltrating NK cells and T cells. Altogether, these data demonstrate that the inhibitory effect of ammonia on cytotoxicity and perforin levels is universal for both NK and T cells and occurs *in vivo*.

## Discussion

NK and T-cell–based immunotherapies are currently extensively studied in preclinical and clinical studies (3, 44). This field currently involves CAR-based therapies, monoclonal antibodies with modified Fc regions to enhance ADCC, as well as bi- and trispecific NK and T-cell–engaging antibodies. Nonetheless, the TME remains one of the main obstacles to the effectiveness of NK and T-cell–based therapy (5, 45). In this study, we identified that ammonia in the TME is a metabolite that inhibits the cytotoxicity of NK and T cells. We demonstrated that cancer-conditioned medium suppressed the antitumor activity of NK cells. Ammonia accumulated in the conditioned medium and TME, reaching concentrations from 2 to 5 mmol/L, which is similar to previously reported values (11, 46, 47). At these concentrations, ammonia inhibited the cytotoxicity of NK cells and CAR T cells and decreased mature perforin levels, leading to NK/CAR T-cell dysfunction (Fig. 7K).

Recently, ammonia has emerged as an important player in cancer. Although systemic hyperammonemia in patients with cancer is rather rare and generally does not exceed micromolar concentrations (48), ammonia accumulates in the TME reaching millimolar concentrations in consequence of the breakdown of glutamate by glutaminase (22). In cancer, ammonia is recycled and serves as a source of nitrogen to build amino acids, promote the growth of tumor biomass, support autophagy, and protect cells from TNFα-induced cell death (11, 46, 49). Conversely, at high concentrations, ammonia suppresses polyamine biosynthesis and inhibits cancer cell proliferation (50). Ammonia can be utilized by three enzymes: carbamoyl phosphate synthase-1, glutamine synthetase, and glutamate dehydrogenase, which are regulated by oncogenes and growth signaling pathways (51). Low expression of ammonia-detoxifying carbamoyl phosphate synthase-1 is associated with worse overall survival of patients with hepatocellular cancer (52). A high ammonia gene expression signature, which includes low expression of ammonia-detoxifying enzymes and high expression of ammonia-producing enzymes, is associated with decreased survival of patients with gastrointestinal carcinomas (16). Notably, other types of cells in the TME also contribute to nitrogen metabolism and, thus, the production of ammonia (22, 51).

Furthermore, ammonia promotes cancer progression indirectly by suppressing the antitumor immune response. A recent study demonstrated that ammonia inhibits T-cell–dependent antitumor immunity and enhances their exhaustion (16). The gene signature associated with high ammonia was a predictor of worse survival following immune checkpoint inhibitors (16). Moreover, ammonia inhibits antigen presentation by macrophages and phagocytosis by neutrophils and impairs the functions of dendritic cells (12–15). In this study, we demonstrated that the cytotoxicity of both NK and T cells is also impaired by ammonia, thus suggesting its wide suppressive effects on the immune response.

It is well established that cancer cells and tumor-promoting immune cells suppress the antitumor activity of both NK and T cells. Different cytokines, including TGFβ, IL6, and IL10, as well as cancer-associated metabolites, including tryptophan metabolites, lactate, and prostaglandin E2, inhibit cytotoxic cell functions at various levels (5). These factors regulate the expression of chemokine receptors, activation and inhibitory receptors, cytokines, and molecules of the lytic granule: granzymes and perforin.

Perforin has a primary role in the NK cell–mediated control of tumor initiation, growth, and metastasis (53, 54). It is necessary for granule-dependent target cell death because it enables granzyme-induced apoptosis (31). Without perforin, NK cells are unable to perform granzyme B–mediated serial killing and only kill via death receptors, which significantly prolongs the time required to kill cancer cells (41, 55). In this study, we observed that ammonia significantly impairs the antitumor activity of NK cells by decreasing perforin. Perforin is synthesized as a 70 kDa inactive precursor that is subjected to proteolytic cleavage and different modifications to yield a mature 60 kDa active form (27). Trafficking of perforin from the endoplasmic reticulum through the Golgi to the lysosomal compartment is accompanied by LAMP1/CD107a (56). NK cells with LAMP1 knockdown have impaired perforin recruitment to lytic granules, which leads to the inhibition of their cytotoxicity (56). The activity of mature perforin is regulated by two main factors: pH and calcium ions (31). Moreover, mature perforin is stored in lysosomal granules (secretory lysosomes), in which low pH prevents oligomerization, thus protecting cytotoxic lymphocytes (57). In secretory lysosomes, the lytic activity of perforin is prevented by the chaperon protein calreticulin and proteoglycans (58, 59). This association of perforin with granule proteins is pH-dependent, and increased pH leads to the dissociation of perforin from the proteoglycans (59, 60). In this study, we demonstrated that ammonia increases pH in the lysosomes and decreases the amount of mature perforin. Previous studies demonstrated that alkalization of the acidic compartment impairs perforin maturation by inhibiting proteolytic cleavage (27), leading to its inactivation and proteolytic degradation (61). In our study, we observed the rapid disappearance of mature perforin in low concentrations of ammonia and a decrease in immature perforin upon treatment with 4 to 5 mmol/L ammonia (Fig. 5E). This may suggest that its proteolytic degradation plays a role in the effects exerted by ammonia on NK cells. Our results with enzymatic cleavage in cell-free assays show that both cathepsin L and granzyme B degrade recombinant mature perforin. Therefore, we hypothesize that by increasing pH, ammonia releases perforin from interaction with proteoglycans and makes it easily accessible for degradation. Interestingly, lysosomal pH neutralization affects neither the amount of granzyme B nor granzyme H. We cannot exclude the possibility that no proteases in the secretory lysosomes exist that could cleave and thereby inactivate granzymes. Conceptually, it is logical that specific mechanisms exist that inactivate perforin only. Perforin is the most dangerous substance in the granules as it could form pores in intracellular membranes and potentially kill the NK/T cell from within. Active granzymes in the granules would not be dangerous to the cytotoxic cells as granzymes alone could not escape the vesicles. It is also possible, but requires further extensive studies, that active granzymes constitute one of the mechanisms protecting T/NK cells from perforin activation within the effector cells. In the presence of ammonia, NK cells compensatorily upregulated the expression of death receptors (Fig. 6C and D). Nonetheless, they still had impaired cytotoxic functions as they needed significantly more time to kill target cells (Fig. 6G), and the majority of their contacts with target cells did not lead to successful killing (Fig. 6I). Furthermore, although nearly all tumor cells respond to granule-mediated cytotoxicity, the induction of apoptosis via death receptors relies on the presence of CD95/Fas or TRAILR1/TRAILR2 on the tumor cell surface. As a result, shifting from granule-mediated to death receptor–mediated cytotoxicity will not lead to the destruction of tumors lacking these death receptors, thereby restricting the range of tumor cells that are vulnerable to the cytotoxic effects of T and NK cells. In contrast, it could even induce fratricide killing of neighboring NK or T cells. Previous studies demonstrated that defective killing results in enhanced cytokine secretion, which is caused by failed detachment from target cells (24, 55). Indeed, we observed that ammonia increased levels of IFNγ and TNFα in NK cells (Fig. 4D). Such delayed detachment was observed in human NK cells with genetic perforin knockout (24). Accordingly, we observed that ammonia delayed the disengagement of NK cells from their targets (Fig. 4E). Notably, previous studies revealed that ammonia does not affect target cell sensitivity to killing by purified granzyme B and perforin (62), further confirming the selective impairment of NK cell cytotoxic machinery by ammonia. We hypothesize that the consequences of exposure of T/NK cells to ammonia could be similar to those observed in patients with familial hemophagocytic lymphohistiocytosis, characterized by complete loss of perforin activity due to perforin gene mutations (63). In patients with familial hemophagocytic lymphohistiocytosis, the clinical manifestations reflect both cytokine hypersecretion (due to chronic stimulation of T and NK cells, resulting in disordered immune hemostasis affecting both the lymphoid and myeloid compartments) and failure to clear dangerous target cells, particularly virus-infected cells and neoplastic cells.

It was already shown by others that ammonia could be an important player shaping the immune landscape within the tumor (15–18). In this study, we add another piece to understanding how microenvironmental ammonia drives the dysfunction of T/NK cells. Our results are further confirmed by an analysis of a published proteomic dataset from patients with HCC (20), revealing that perforin levels are decreased in tumor-infiltrating NK cells and T cells compared with their blood counterparts. We cannot exclude that in this study, the decrease in perforin is to some extent a result of the degranulation of tumor-infiltrating NK cells and T cells in contact with target tumor cells. Therefore, we also performed an *in vivo* study in a murine model in which NK/CAR T cells were intratumorally injected into Raji tumor–bearing mice. In these experiments, perforin levels in cytotoxic cells isolated from tumor masses were compared with control NK/CAR T cells that were exposed to target tumor cells *in vitro* in control media. We observed a significant decrease in perforin in cytotoxic cells from tumor masses, further confirming that this effect is induced by the TME. Importantly, different components of the TME, including hypoxia, PGE2, lactate, and kynurenines, decrease perforin levels in NK cells (64–66). However, the underlying molecular mechanisms remain unknown. In this study, we identified that ammonia, even at low concentrations, potently decreases the amount of mature perforin and severely impairs perforin-/granzyme B–mediated killing. Although ammonia itself does not change the pH of the media, it specifically alkalizes cellular acidic compartments and is therefore referred to as a lysosomotropic agent. Ammonia (NH_3_) is neutral and rapidly passes across biological membranes. In contrast, ammonium ions (NH_4_^+^) cannot diffuse through membranes and accumulate in acidic organelles, including lysosomes. A recent study identified SLC12A9 as a lysosome-detoxifying ammonium chloride cotransporter (67). However, its expression in immune cells remains unknown. Moreover, although hepatocytes and tumor cells can metabolize ammonia, immune cells such as T cells, macrophages, and NK cells lack this feature (Supplementary Fig. S4B; ref. 16), which may explain its immunosuppressive effects. Recent studies demonstrated that CD8 memory (T_mem_) cells, throughout their development, gain the ability to detoxify ammonia (17, 18, 68), which explains why CAR T cells are relatively less sensitive to the inhibitory effects of ammonia than NK cells. However, whether some populations of NK cells possess that feature remains unknown. Still, metabolic impairment is one of the key factors driving NK and T-cell dysfunction in tumors, and reprogramming of metabolic fitness can strengthen their antitumor activity (69). Therapeutic strategies that decrease ammonia in the TME may restore NK cell and T-cell activity against the tumors. This is supported by the fact that the effects of low ammonia concentrations on the level of perforin are fully reversible (Fig. 5C). A previous study demonstrated that neutralization of ammonia reduced colorectal cancer tumor growth and enhanced T-cell response to immune checkpoint inhibitors (16). Moreover, there are other strategies to modulate ammonia levels in the TME. A nonpathogenic strain of *Escherichia coli* was engineered to use ammonia for continuous arginine synthesis (70, 71). This strain recycles ammonia and elevates the arginine amount necessary for T cells in the TME, leading to the activation of the T-cell response, inhibition of tumor growth, and prolonged survival of tumor-bearing mice (70, 72). On the other hand, a high ammonia level may be used as a marker of the TME to selectively deliver different cytotoxic compounds to tumors (47). Moreover, a better understanding of the mechanisms involved in the degradation of perforin upon alkalization of secretory lysosomes could help design strategies aimed at reactivating NK and T cells in the TME.

## Supplementary Material

Supplementary Fig. 1 Supplementary Fig. 1 shows that culture-conditioned medium suppresses cytotoxicity of NK cells

Supplementary Fig. 2 Supplementary Fig. 2 shows that low molecular fraction of conditioned medium suppresses cytotoxicity of NK cells

Supplementary Fig. 3 Supplementary Fig. 3 shows changes in pH and ammonia concentration in conditioned media

Supplementary Fig. 4 Supplementary Fig. 4 shows that ammonia accumulates in NK cells, affects their cytotoxic potential but not their viability.

Supplementary Fig. 5 Supplementary Fig. 5 shows ammonium chloride’s impact on the efficiency of rituximab therapy in vivo

Supplementary Fig. 6 Supplementary Fig. 6 shows that ammonia is rapidly excreted or metabolized in TME

Supplementary Fig. 7 Supplementary Fig. 7 shows that conditioned medium decreases expression and secretion of perforin by NK cells

Supplementary Fig. 8 Supplementary Fig. 8 shows effects of ammonia on perforin gene expression at the transcriptional level

Supplementary Fig. 9 Supplementary Fig. 9 shows that ammonia decreases acidic compartment in NK cells

Supplementary Fig. 10 Supplementary Fig. 10 shows that ammonia increases pH in the lysosomes of NK-92 cells.

Supplementary Fig. 11 Supplementary Fig. 11 shows that ammonia increases the number of LAMP1+ vesicles but decreases their volume and LAMP1 level

Supplementary Fig. 12 Supplementary Fig. 12 shows lack of impact of cycloheximide (CHX), a compound blocking protein translation, on mature perforin levels

Supplementary Fig. 13 Supplementary Fig. 13 shows the effects of ammonia on T cells.
