# Supplementary material for: Ammonia Suppresses the Antitumor Activity of Natural Killer Cells and T Cells by Decreasing Mature Perforin
Source: Cancer Res. 2025 Mar 31;85(13):2448–67. doi: 10.1158/0008-5472.CAN-24-0749 (PMC12214879; doi:10.1158/0008-5472.CAN-24-0749)
Supplement: Supplementary Fig. 1 — shows that culture-conditioned medium suppresses cytotoxicity of NK cells [file can-24-0749_supplementary_fig.1_suppsf1.docx]

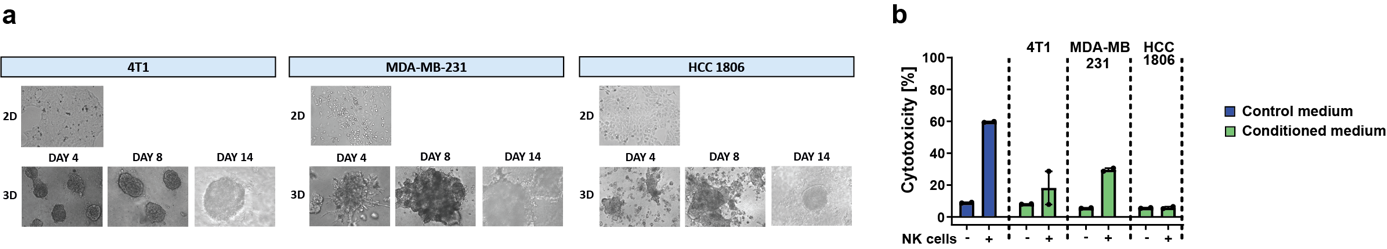


**Supplementary Fig. 1. 3D cancer culture-conditioned medium suppresses cytotoxicity of NK cells**

**a**, Microscopy photographs of 4T1, MDA-MB-231 and HCC 1806 cells cultured in conventional conditions (2D) and three-dimensional culture (3D). Conditioned medium was collected on the 14^th^ day after 48h of culture. **b**, Natural cytotoxicity of NK cells against K562 cells in the presence of control medium and breast cancer 3D culture-conditioned medium. Data from a representative experiment. K562 cells were stained with CFSE and incubated with NK cells in a medium conditioned by indicated cells. Cytotoxicity was assessed after 4h using flow cytometry and presented as percentage of propidium iodide-positive CFSE-positive (K562) cells.
