# Supplementary material for: Ammonia Suppresses the Antitumor Activity of Natural Killer Cells and T Cells by Decreasing Mature Perforin
Source: Cancer Res. 2025 Mar 31;85(13):2448–67. doi: 10.1158/0008-5472.CAN-24-0749 (PMC12214879; doi:10.1158/0008-5472.CAN-24-0749)
Supplement: Supplementary Fig. 2 — shows that low molecular fraction of conditioned medium suppresses cytotoxicity of NK cells [file can-24-0749_supplementary_fig.2_suppsf2.docx]

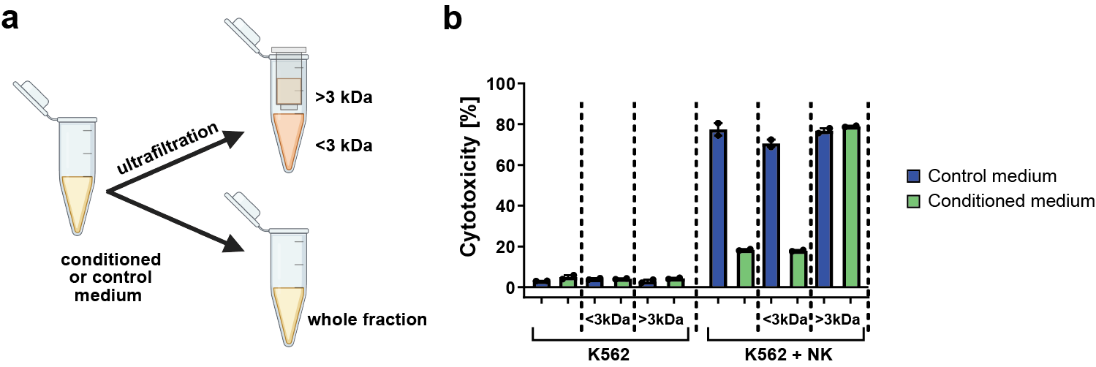


**Supplementary Fig. 2. Low molecular fraction of conditioned medium suppresses cytotoxicity of NK cells**

**a**, Raji-cells conditioned medium was divided using an ultrafiltration method into a low molecular fraction (< 3kDa) and a higher molecular fraction (> 3kDa). Created in BioRender. Winiarska, M. (2025) <https://BioRender.com/r43f195> **b**, Natural cytotoxicity of NK cells against K562 cells in the presence of the whole fraction, low molecular fraction (<3 kDa) and higher molecular fraction (> 3 kDa) of control and Raji cells-conditioned medium. Data from a representative experiment. K562 cells were stained with CFSE and incubated with NK cells in a medium conditioned by indicated cells. Cytotoxicity was assessed after 4h using flow cytometry and plotted as percentage of propidium iodide-positive CFSE-positive (K562) cells.
