# Supplementary material for: Ammonia Suppresses the Antitumor Activity of Natural Killer Cells and T Cells by Decreasing Mature Perforin
Source: Cancer Res. 2025 Mar 31;85(13):2448–67. doi: 10.1158/0008-5472.CAN-24-0749 (PMC12214879; doi:10.1158/0008-5472.CAN-24-0749)
Supplement: Supplementary Fig. 3 — shows changes in pH and ammonia concentration in conditioned media [file can-24-0749_supplementary_fig.3_suppsf3.docx]

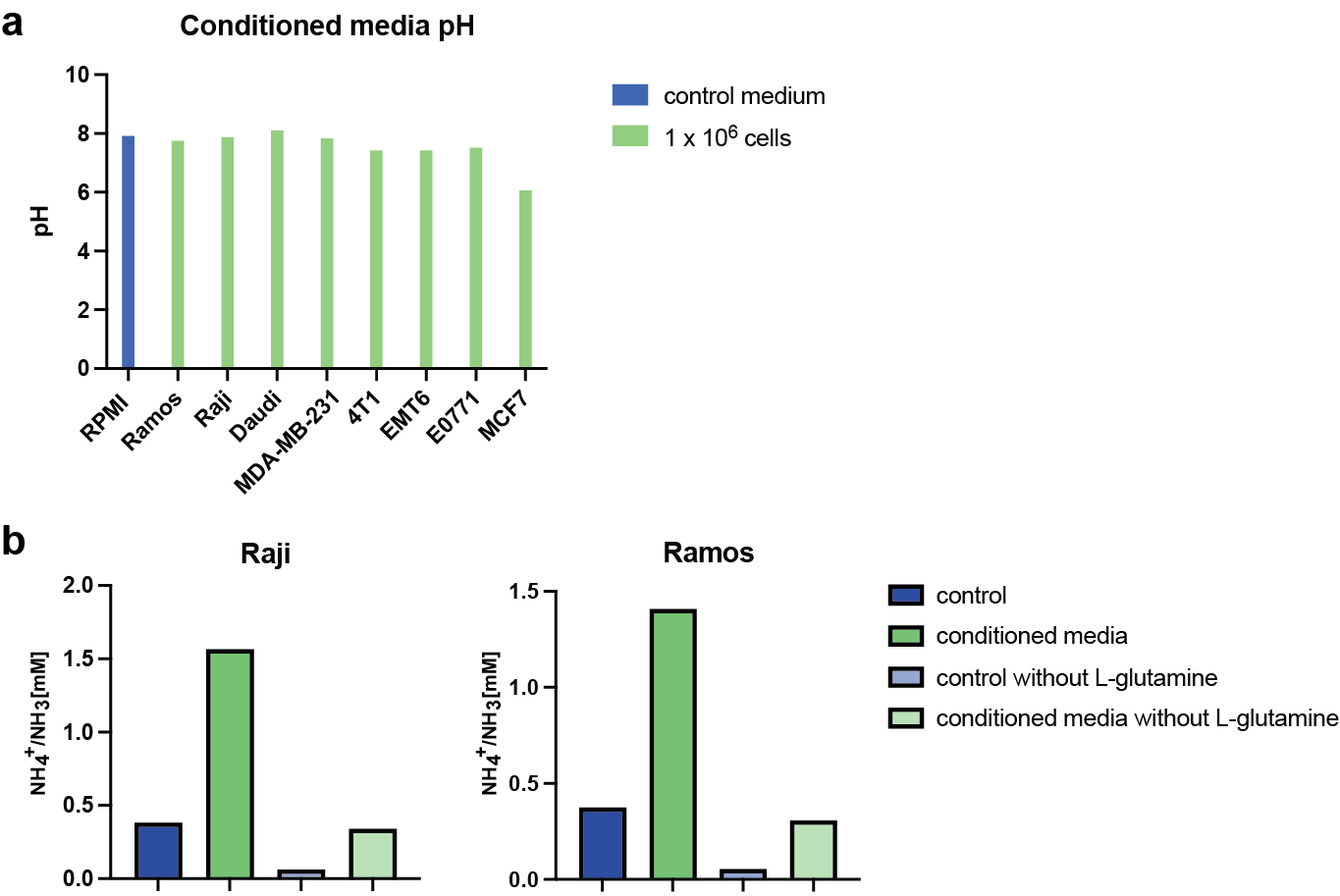


**Supplementary Fig. 3 Changes in pH and ammonia concentration in conditioned media.**

**a,** Media was conditioned using different cancer cell lines followed by measurement of the pH (n=1). **b,** Control medium or medium without L-glutamine was used to generate Raji- or Ramos-conditioned media. The concentration of ammonia was measured using a Dimension Ammonia assay (Siemens) (n=1). Data related to Fig. 1f.
