# Supplementary material for: Ammonia Suppresses the Antitumor Activity of Natural Killer Cells and T Cells by Decreasing Mature Perforin
Source: Cancer Res. 2025 Mar 31;85(13):2448–67. doi: 10.1158/0008-5472.CAN-24-0749 (PMC12214879; doi:10.1158/0008-5472.CAN-24-0749)
Supplement: Supplementary Fig. 4 — shows that ammonia accumulates in NK cells, affects their cytotoxic potential but not their viability. [file can-24-0749_supplementary_fig.4_suppsf4.docx]

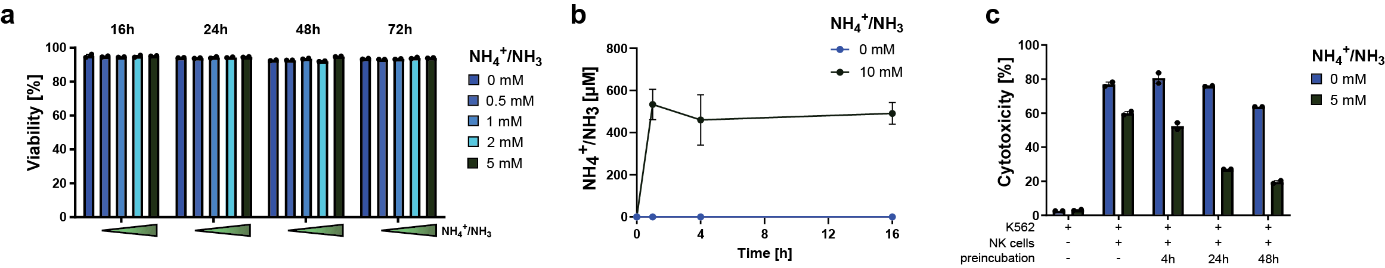


**Supplementary Fig. 4. Ammonia accumulates in NK cells, affects their cytotoxic potential but not their viability.**

**a**, Viability of NK cells incubated with different concentrations of ammonia (ammonium chloride) for 16, 24, 48, and 72h was assessed using propidium iodide staining and flow cytometry (n=2). **b,** The concentration of ammonia in NK cells after incubation with ammonia (ammonium chloride) for 30 min, 4 and 16h (n=3). After the indicated time, cells were washed two times with PBS and lysed. **c**, Natural cytotoxicity of NK cells against K562 cells in the presence of ammonia (ammonium chloride) (n=2). Cytotoxicity was assessed after 4h using flow cytometry and determined as percentage of propidium iodide-positive CFSE-positive (K562) cells. NK cells were preincubated with ammonia for 4, 24, or 48h, as indicated in the figure.
