# Supplementary material for: Ammonia Suppresses the Antitumor Activity of Natural Killer Cells and T Cells by Decreasing Mature Perforin
Source: Cancer Res. 2025 Mar 31;85(13):2448–67. doi: 10.1158/0008-5472.CAN-24-0749 (PMC12214879; doi:10.1158/0008-5472.CAN-24-0749)
Supplement: Supplementary Fig. 5 — shows ammonium chloride’s impact on the efficiency of rituximab therapy in vivo [file can-24-0749_supplementary_fig.5_suppsf5.docx]

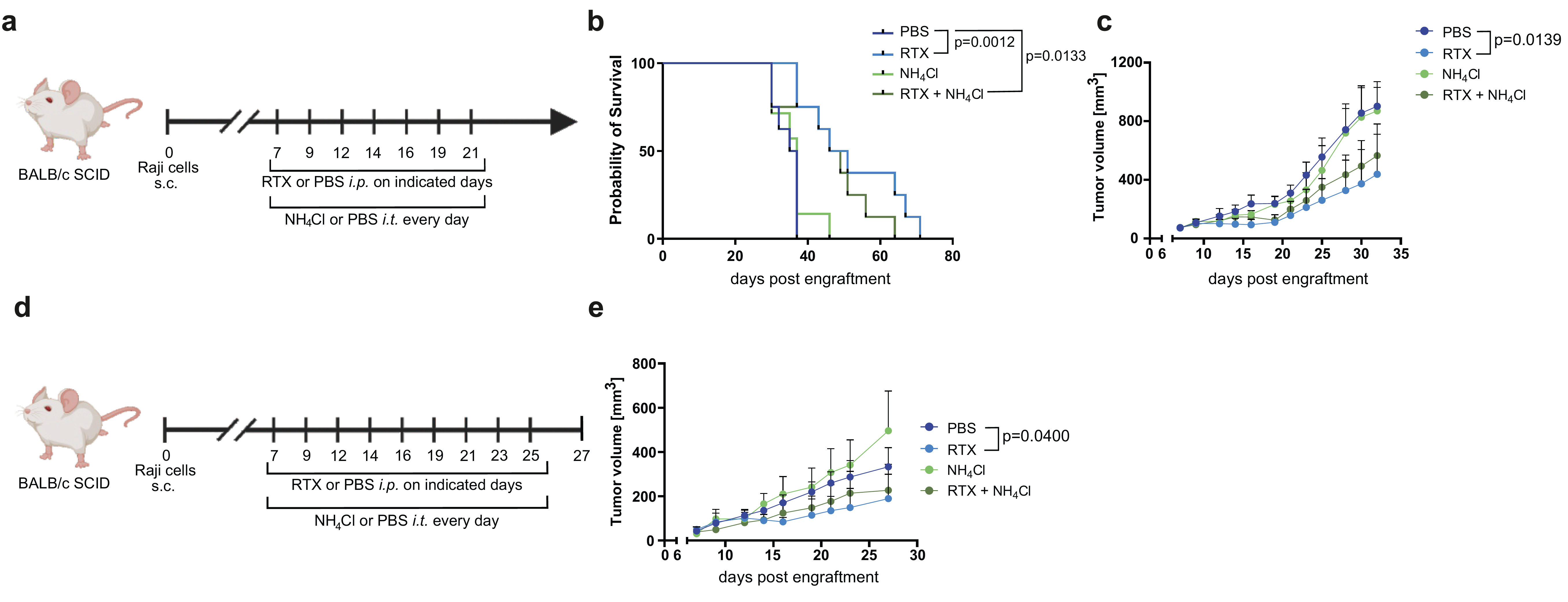


**Supplementary Fig. 5. Ammonium chloride’s impact on the efficiency of rituximab therapy *in vivo***

**a**, In vivo experimental scheme created in BioRender. Winiarska, M. (2025) https://BioRender.com/y35q855. Raji cells were injected into BALB/c mice. 7 days after injection, mice started receiving rituximab (RTX) or PBS intraperitoneally (*i.p.*) and were injected intratumorally (*i.t.*) with either ammonia (ammonium chloride) or PBS for 14 days. **b-c**, Survival (**b**), and tumor growth (**c**) of mice in each group (n=5 mice/group). **d**, In vivo experimental scheme created in BioRender. Winiarska, M. (2025) https://BioRender.com/y35q855. Raji cells were injected into BALB/c mice. 7 days after injection, mice started receiving RTX or PBS (*i.p.*) and were injected *i.t.* with either ammonia (ammonium chloride) or PBS for 18 days. **e,** the graph shows the tumor growth (**c**) of mice in each experimental group (n=5 mice/group).
