# Supplementary material for: Ammonia Suppresses the Antitumor Activity of Natural Killer Cells and T Cells by Decreasing Mature Perforin
Source: Cancer Res. 2025 Mar 31;85(13):2448–67. doi: 10.1158/0008-5472.CAN-24-0749 (PMC12214879; doi:10.1158/0008-5472.CAN-24-0749)
Supplement: Supplementary Fig. 6 — shows that ammonia is rapidly excreted or metabolized in TME [file can-24-0749_supplementary_fig.6_suppsf6.docx]

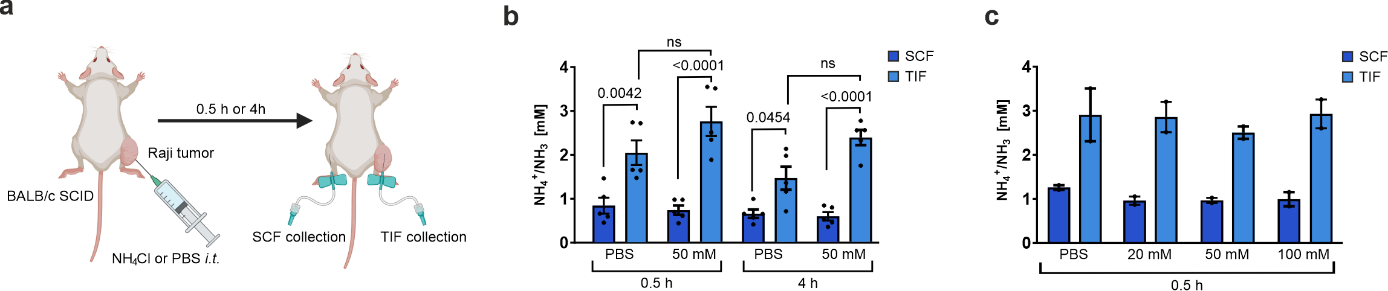


**Supplementary Fig. 6. Ammonia is rapidly excreted or metabolized in TME**

**a**, *In vivo* experimental scheme. Raji cells were injected into BALB/c mice. 7 days after injection, mice were injected *i.t.* with either ammonia (ammonium chloride) or PBS. After 30 min or 4h TIF and SCF were collected followed by an ammonia measurement. Created in BioRender. Winiarska, M. (2025) https://BioRender.com/x56a982 **b-c**, Concentration of ammonia in TIF and SCF isolated from Raji tumors after *i.t.* administration of ammonia (ammonium chloride) or PBS (**b,** n=5; **c**, n=2).
