# Supplementary material for: Ammonia Suppresses the Antitumor Activity of Natural Killer Cells and T Cells by Decreasing Mature Perforin
Source: Cancer Res. 2025 Mar 31;85(13):2448–67. doi: 10.1158/0008-5472.CAN-24-0749 (PMC12214879; doi:10.1158/0008-5472.CAN-24-0749)
Supplement: Supplementary Fig. 7 — shows that conditioned medium decreases expression and secretion of perforin by NK cells [file can-24-0749_supplementary_fig.7_suppsf7.docx]

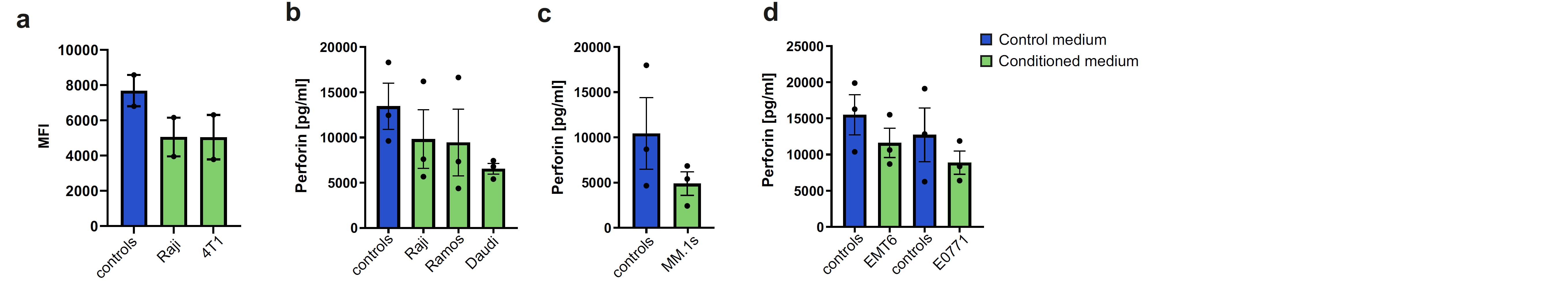


**Supplementary Fig. 7. Conditioned medium decreases expression and secretion of perforin by NK cells**

**a**, The level of perforin detected in NK cells incubated with Raji- or 4T1-conditioned medium determined by intracellular staining using anti-perforin antibody (δG9 clone) and flow cytometry (n=2). **b-d,** The concentration of extracellular perforin secreted by NK cells in response to contact with target cells (K562) in **b**, lymphoma cells-conditioned medium (n=3), **c,** multiple myeloma cells-conditioned medium (n=3), and **d,** breast cancer cells-conditioned medium (n=3).
