# Supplementary material for: Ammonia Suppresses the Antitumor Activity of Natural Killer Cells and T Cells by Decreasing Mature Perforin
Source: Cancer Res. 2025 Mar 31;85(13):2448–67. doi: 10.1158/0008-5472.CAN-24-0749 (PMC12214879; doi:10.1158/0008-5472.CAN-24-0749)
Supplement: Supplementary Fig. 8 — shows effects of ammonia on perforin gene expression at the transcriptional level [file can-24-0749_supplementary_fig.8_suppsf8.docx]

**
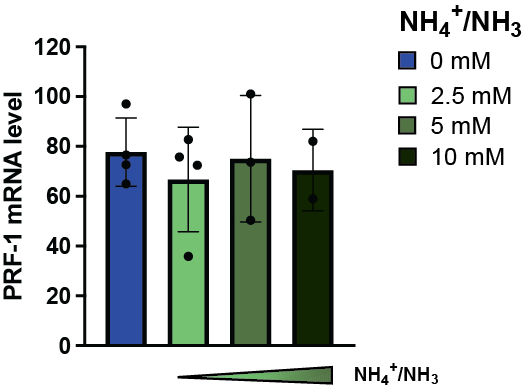
**

**Supplementary Fig. 8. Effects of ammonia on perforin gene expression at the transcriptional level.**

PRF-1 mRNA level in NK cells after treatment with ammonia (NH_4_Cl) for 24 h. PRF-1 mRNA level was calculated using the ΔCt method relative to the mean of TBP and SDHA housekeeping genes expression. Data show mean ± SD from 2-4 donors performed in 3 technical replicates each. For statistical analysis, one-way ANOVA with Dunnett’s post-hoc test for multiple comparisons were used. No significant differences were detected.
