# Supplementary material for: Ammonia Suppresses the Antitumor Activity of Natural Killer Cells and T Cells by Decreasing Mature Perforin
Source: Cancer Res. 2025 Mar 31;85(13):2448–67. doi: 10.1158/0008-5472.CAN-24-0749 (PMC12214879; doi:10.1158/0008-5472.CAN-24-0749)
Supplement: Supplementary Fig. 9 — shows that ammonia decreases acidic compartment in NK cells [file can-24-0749_supplementary_fig.9_suppsf9.docx]

**
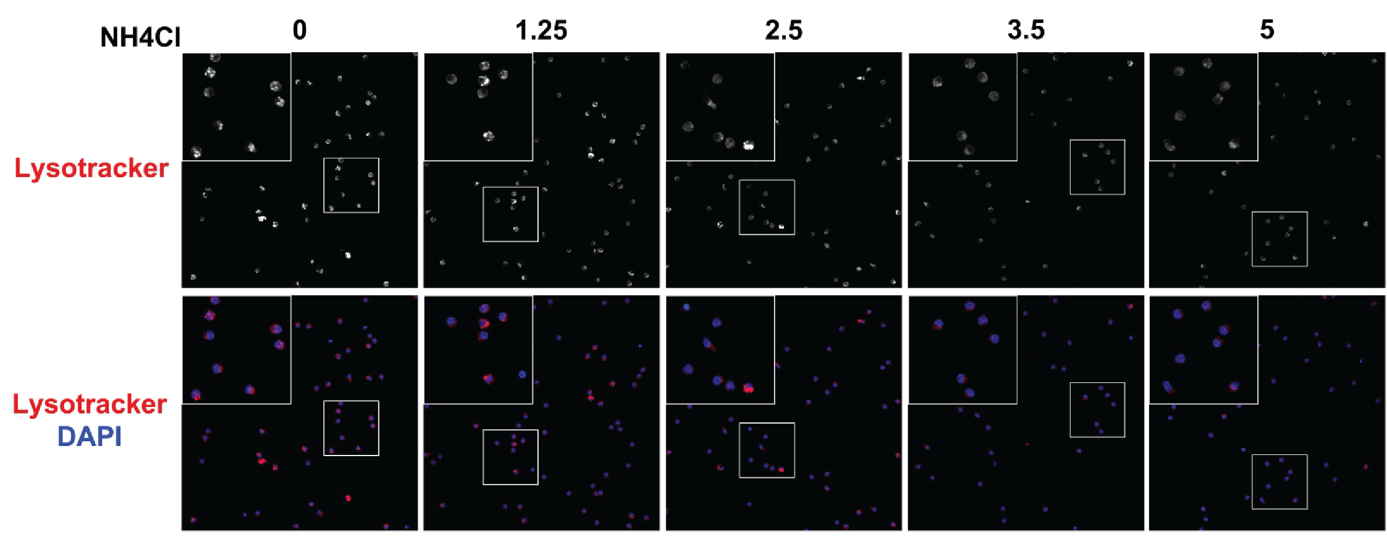
**

**Supplementary Fig. 9. Ammonia decreases acidic compartment in NK cells.**

The lysosomal content of NK cells incubated with ammonia (ammonium chloride) for 12h. Acidic organelles were stained using a LysoTracker and live imaged using Opera Phenix cell microscopy (n=4).
