# Supplementary material for: Ammonia Suppresses the Antitumor Activity of Natural Killer Cells and T Cells by Decreasing Mature Perforin
Source: Cancer Res. 2025 Mar 31;85(13):2448–67. doi: 10.1158/0008-5472.CAN-24-0749 (PMC12214879; doi:10.1158/0008-5472.CAN-24-0749)
Supplement: Supplementary Fig. 10 — shows that ammonia increases pH in the lysosomes of NK-92 cells. [file can-24-0749_supplementary_fig.10_suppsf10.docx]

**
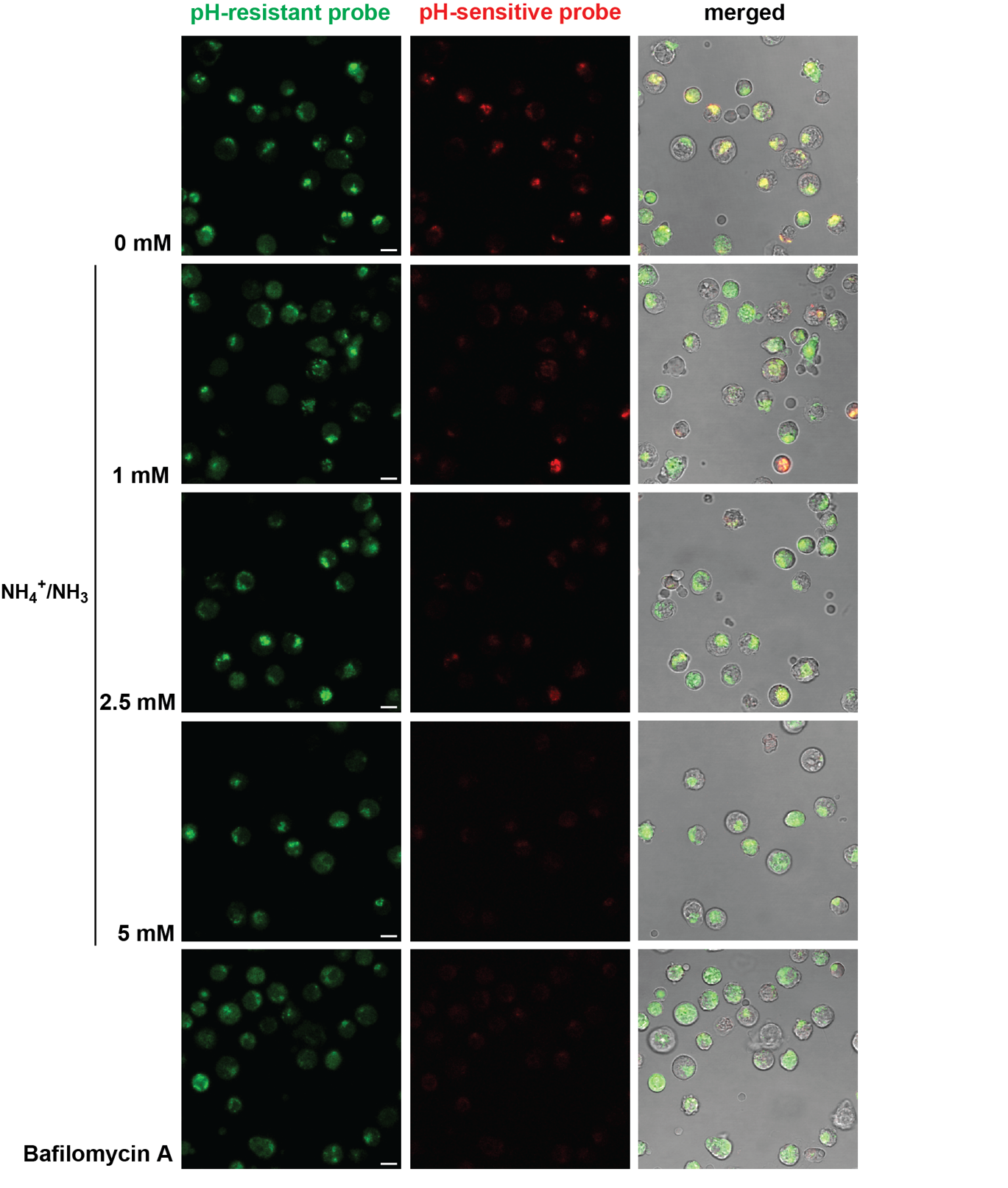
**

**Supplementary Fig. 10. Ammonia increases pH in the lysosomes of NK-92 cells.**

The cells were loaded with the lysosomal fluorescent probes LysoPrime Green (the pH-resistant probe) and pHLys Red (the pH-sensitive probe; Dojindo Laboratories) and treated with increasing concentrations of NH_4_Cl (from 0 to 5 mM) or bafilomycin A as a control in the imaging medium. Staining with LysoPrime Green is shown in the left column (green; the scale bar denotes 10 um), staining with pHLys Red is shown in the middle column (red), and the merge of LysoPrime Green signal, pHLys Red signal and transmitted light image (grey) is shown in the right column. Data related to Fig. 5h. (n=3).
