# Supplementary material for: Ammonia Suppresses the Antitumor Activity of Natural Killer Cells and T Cells by Decreasing Mature Perforin
Source: Cancer Res. 2025 Mar 31;85(13):2448–67. doi: 10.1158/0008-5472.CAN-24-0749 (PMC12214879; doi:10.1158/0008-5472.CAN-24-0749)
Supplement: Supplementary Fig. 11 — shows that ammonia increases the number of LAMP1+ vesicles but decreases their volume and LAMP1 level [file can-24-0749_supplementary_fig.11_suppsf11.docx]

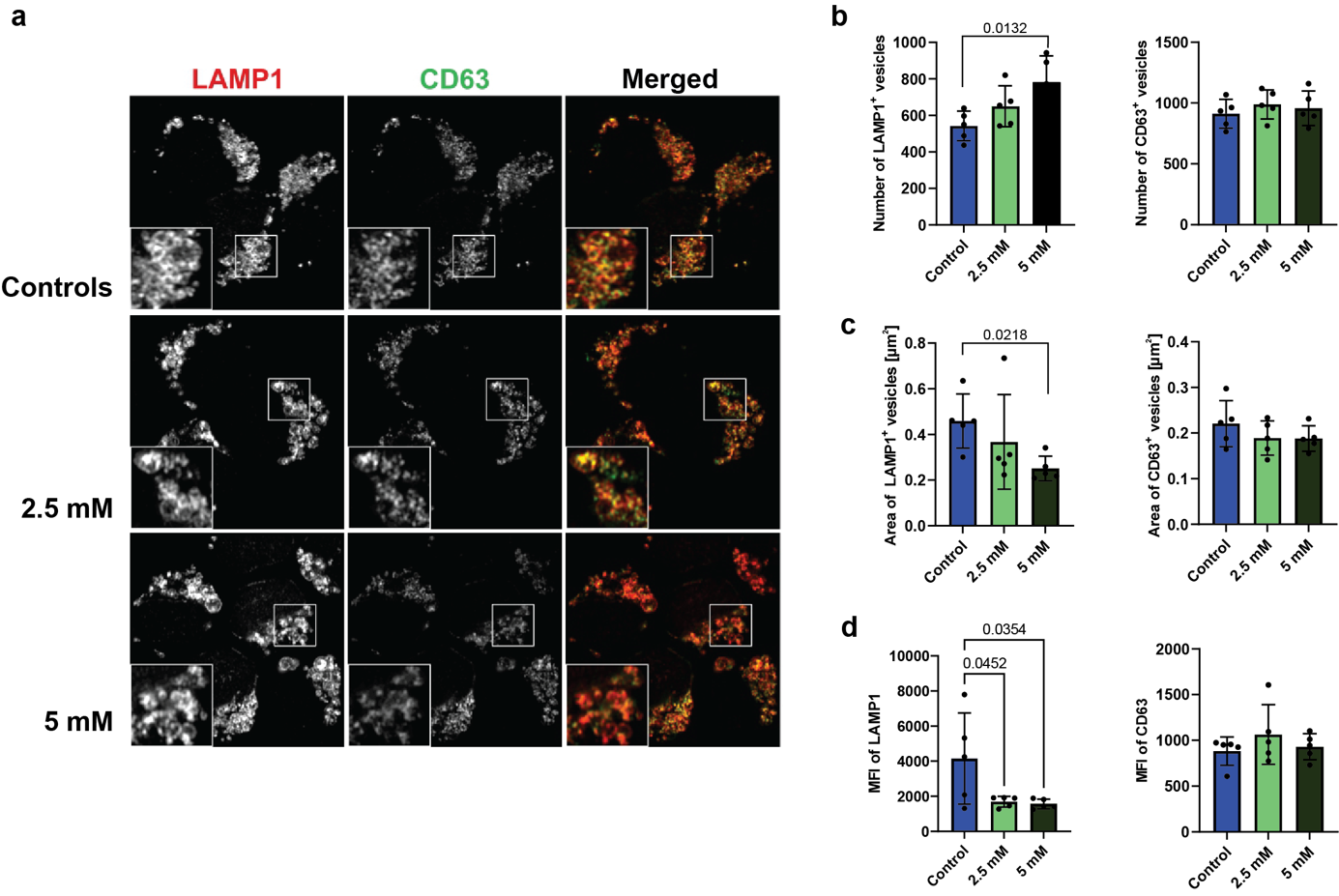
**Supplementary Fig. 11. Ammonia increases the number of LAMP1^+^ vesicles but decreases their volume and LAMP1 level**

**a,** LAMP1 and CD63 staining of NK cells incubated in different concentrations of NH_4_Cl. Cells were incubated with NH_4_Cl for 4h, followed by antibody staining and imaging using ZEISS LSM 800 with motion tracking. **b-c,** Number (**b**) and area (**c**) of LAMP1^+^ and CD63^+^ vesicles in a single NK cell incubated with different concentrations of NH_4_Cl (n=5). **d,** Mean fluorescence intensity (MFI) of LAMP1 and CD63 staining in a single NK cell incubated with different concentrations of NH_4_Cl (n=5).
