# Supplementary material for: Ammonia Suppresses the Antitumor Activity of Natural Killer Cells and T Cells by Decreasing Mature Perforin
Source: Cancer Res. 2025 Mar 31;85(13):2448–67. doi: 10.1158/0008-5472.CAN-24-0749 (PMC12214879; doi:10.1158/0008-5472.CAN-24-0749)
Supplement: Supplementary Fig. 12 — shows lack of impact of cycloheximide (CHX), a compound blocking protein translation, on mature perforin levels [file can-24-0749_supplementary_fig.12_suppsf12.docx]

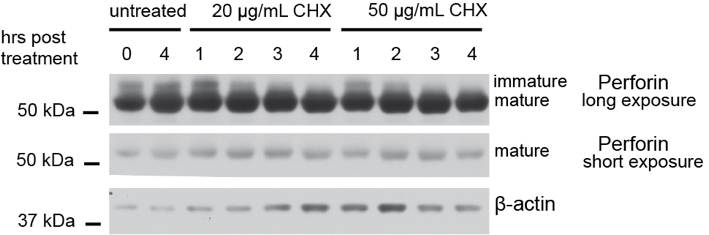


**Supplementary Fig. 12 Lack of impact of cycloheximide (CHX), a compound blocking protein translation, on mature perforin levels.**

NK cells were treated for 4h with cycloheximide (CHX) in the indicated concentrations. Cell lysates were analyzed for perforin expression (Pf-344 clone). Actin levels were determined as loading control. Data from one representative experiment shown (n=3).
