# Supplementary material for: Ammonia Suppresses the Antitumor Activity of Natural Killer Cells and T Cells by Decreasing Mature Perforin
Source: Cancer Res. 2025 Mar 31;85(13):2448–67. doi: 10.1158/0008-5472.CAN-24-0749 (PMC12214879; doi:10.1158/0008-5472.CAN-24-0749)
Supplement: Supplementary Fig. 13 — shows the effects of ammonia on T cells. [file can-24-0749_supplementary_fig.13_suppsf13.docx]

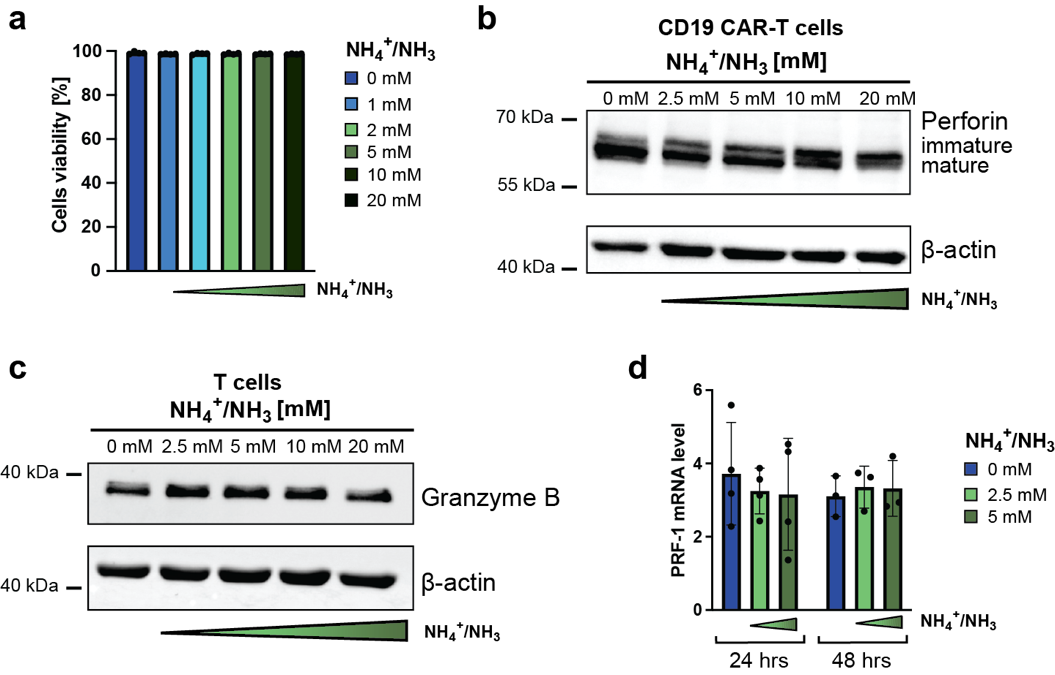


**Supplementary Fig. 13 Effects of ammonia on T cells.**

**a,** Viability of CD19 CAR-T cells incubated with different concentrations of NH_4_Cl 24 was assessed using propidium iodide staining and flow cytometry (n=4). **b**, The level of perforin in CD19 CAR-T cells incubated with NH_4_Cl for 4h determined by Western blot method using anti-perforin antibody (Pf-344 clone) (n=3). **c**, The level of granzyme B in T cells stimulated with anti-CD3/CD28 incubated with NH_4_Cl for 4h determined by Western blot method using anti-granzyme B antibody (M3304B06 clone) (n=3). β-actin presented as a loading control. Representative blot from one donor. **d**, PRF-1 in activated T cells after treatment with NH_4_Cl for 24h and 48h (T cells). PRF-1 mRNA level was calculated using the ΔCt method relative to the mean of TBP and SDHA housekeeping gene expression.
